# Design of Nanoparticles in Cancer Therapy Based on Tumor Microenvironment Properties

**DOI:** 10.3390/pharmaceutics14122708

**Published:** 2022-12-03

**Authors:** Bita Mahdavi Firouzabadi, Maria Rosa Gigliobianco, Joice Maria Joseph, Roberta Censi, Piera Di Martino

**Affiliations:** 1 School of Pharmacy, University of Camerino, CHiP Chemistry Interdisciplinary Project, Via Madonna delle Carceri, 62032 Camerino, Italy; 2 Percuros B.V., Zernikedreef 8, 2333 CL Leiden, The Netherlands; 3 Dipartimento di Farmacia, Università “G. D’Annunzio” Chieti e Pescara, Via dei Vestini 1, 66100 Chieti, Italy

**Keywords:** tumor microenvironment, nano-systems, nanoparticles, cancer therapy

## Abstract

Cancer is one of the leading causes of death worldwide, and battling cancer has always been a challenging subject in medical sciences. All over the world, scientists from different fields of study try to gain a deeper knowledge about the biology and roots of cancer and, consequently, provide better strategies to fight against it. During the past few decades, nanoparticles (NPs) have attracted much attention for the delivery of therapeutic and diagnostic agents with high efficiency and reduced side effects in cancer treatment. Targeted and stimuli-sensitive nanoparticles have been widely studied for cancer therapy in recent years, and many more studies are ongoing. This review aims to provide a broad view of different nanoparticle systems with characteristics that allow them to target diverse properties of the tumor microenvironment (TME) from nanoparticles that can be activated and release their cargo due to the specific characteristics of the TME (such as low pH, redox, and hypoxia) to nanoparticles that can target different cellular and molecular targets of the present cell and molecules in the TME.

## 1. Introduction

Cancer is one of the principal causes of death all over the world. Generally, cancer occurrence and death rates are speedily rising worldwide; this demonstrates both aging and an increase in the population, along with alterations in the occurrence and spreading of the main risk factors for cancer, which are mostly associated with socioeconomic growth. In 2020, approximately 19.3 million new cancer cases and almost 10.0 million cancer expiries occurred worldwide [1].

Besides surgery, present cancer treatments deeply depend on radiation and chemotherapeutic agents, which also affect “normal” cells and result in toxicity in other organs of the patient’s body. As a result, there is an increasing interest in developing extremely effective therapeutic agents that are able to conquer biological obstacles, differentiate between malignant and normal cells, specifically aim at cancerous parts, and give an “intelligent” response to the cancerous tissue for releasing therapeutic agents in the proper place [2]. The cancerous environment, often referred to as the tumor microenvironment, consists of diverse cellular and non-cellular components with specific properties that make it different from the normal physiological conditions present in normal tissues. These special properties have been widely investigated in past years and can be used for different delivery strategies.

Nanoparticles have been the most significant delivery systems of the past few decades in pharmaceutical sciences due to their special characteristics, such as large surface area, high encapsulation efficiency, and controllable release properties. Modifying nanoparticles’ features to have the most efficient system has always been a challenge for formulation scientists and an ever-growing field of study.

Targeted nanoparticles for cancer treatment have been the subject of many studies in the past decade. Targeted cancer therapy can differentiate the minor alterations between normal and malignant conditions. These kinds of therapies exhibit more effectiveness and fewer undesirable adverse effects than conventional treatments. Conventional and non-specific therapies usually have unwanted properties, such as quick removal of the drug and administration of high doses of the drug, which are usually not economical and highly toxic. Nanoparticles have overcome many obstacles of conventional chemotherapeutics, such as not fully efficient biodistribution, side effects, and further drug resistance [3].

New generations of nanoparticles can be modified in almost every aspect for more efficient delivery of therapeutics. Nanoparticles can encapsulate diverse types of therapeutics, such as small molecule drugs, therapeutic antibodies, genetic material, and imaging agents. They can be chemically designed to be responsive to specific properties of cancerous tissue, such as hypoxia and acidic pH. Additionally, nanoparticles can be modified on their surface by targeting antibodies, aptamers, or other types of cellular ligands for cargo delivery to the desired cells.

It is absolutely necessary for researchers working in the field of cancer therapy to obtain fundamental knowledge about the properties of the tumor microenvironment that can be used for designing novel nanoparticle-based delivery systems that would lead to more efficient methods with improved clinical results. There are several different strategies for targeted cancer therapy, from methods targeting the features of the tumor microenvironment to methods targeting the cellular and molecular receptors present in malignant tissues. In this review, first, a general explanation of the tumor microenvironment and its characteristics is provided, then in each chapter, diverse nano-systems for targeting properties of the TME have been reviewed. The aim is to provide a better view of novel nanoparticle systems that can target the TME and be effective as delivery systems for therapeutic and diagnostic agents in cancer treatment.

## 2. Tumor Microenvironment, a Limitation Turned into an Advantage

Conventional cancer chemotherapeutics usually kill cancer cells directly, and their efficacy is dependent on their access and penetration to cancer cells [4]. However, tumor cells are not the only responsible factors for cancer progression, noncancerous stromal cells are also present and highly interactive in the tumor environment [5]. Stromal cells form the tumor microenvironment (TME), which provides support for the proliferation of cancer cells, assisting the escape from natural and immunological mechanisms for programmed cell death. As reported by Hanahan et al. [6], six hallmarks contribute to the progress of cancer and the formation of the TME by cell signaling. These hallmarks are evading cell death pathways, bypassing growth suppressors, constant proliferation signaling, formation of neovasculature (angiogenesis), initiation of metastasis, and proliferative immortality.

The tumor microenvironment consists of cellular and non-cellular components. The former includes numerous stromal cells, such as cancer-associated fibroblasts (CAFs), endothelial cells (ECs), tumor-infiltrating lymphocytes (TILs), myeloid-derived suppressor cells (MDSCs), and tumor-associated macrophages (TAMs). The latter consists of non-soluble elements, such as the extracellular matrix (ECM), and soluble parts, such as growth factors, several cytokines and chemokines, and metabolites [7,8,9,10]. Two main properties of the TME are hypoxia and acidic pH because of cellular metabolism and nutritional requirements. Cancer cells need a high amount of oxygen to proliferate and grow, and since even angiogenesis cannot provide the excess oxygen for the cancerous tissue, the TME is always in a hypoxic condition. Cancer cells boost glycolysis and other metabolic pathways to stand the insufficient oxygen amounts. Another important property, especially in solid tumors, is the impermeability and difficult penetration of the nanomedicine, which requires further modification in the physical and chemical properties of the nanoparticles to increase their permeability inside the TME [11,12,13].

The TME is fundamentally immunosuppressive to defend tumor cells against immune surveillance. It is also a dynamic environment for supporting quick tumor growth and standing all stress factors, for instance, chemotherapy [14,15]. There is an overexpression of growth factors either on cancer cells or other present cells in the TME to provide the increasing needs of cancer cells. Defections in tumor protein 53 (TP53) and retinoblastoma (RB)-associated pathways (which are responsible for detecting any irregularities and starting the procedure of apoptosis) lead these cells to bypass the apoptotic process. Angiogenesis, another important factor for cancer progression, is essential for the cancerous tissue to provide oxygen for cells and is stimulated by several growth factors, such as vascular endothelial growth factor (VEGF) and fibroblast growth factor (FGF). Due to extreme growth levels and an excessive amount of division sets, two obstacles for replication arise: the cell senescence and crisis, in which the cell moves in a non-replicative yet viable phase, and the cell quantity drops. Cancer cells are described by unlimited cell growth and replication, thus displaying proliferative immortality. The procedure for metastasis and invasion is started after the interconnection of adhesion molecules to the extracellular matrix (ECM). The proteolytic enzymes are released by cells, and they exit the ECM, enter the bloodstream, and then are transported into the body [6].

Overall, these procedures correspond to the formation of the tumor microenvironment responsible for the maintenance and progression of cancer. In fact, the TME provides the best supporting system for cancers to grow. Therefore, combatting TME conditions seems to be a wise approach to cancer therapy.

**Figure 1:**
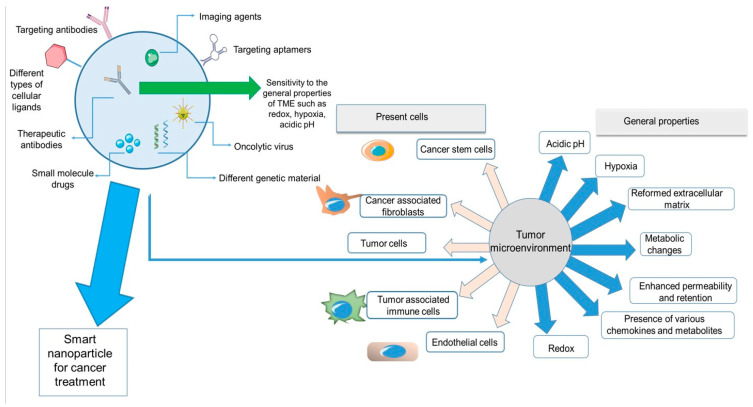
Summary of the properties and components of TME and different features of smart nanoparticles.

Figure 1 depicts a summary of all the properties and components of the tumor microenvironment and nanoparticles discussed in this review. On the left side of the figure, a nanoparticle system is demonstrated. The smart nanoparticle systems can be modified on the surface by diverse cellular ligands and encapsulate different imaging or therapeutic agents for cancer treatment. These systems can have effects on different parts of the TME, from cellular components (cancer cells, immune cells, stem cells, and fibroblasts) to the general characteristics of the TME (hypoxia, redox, metabolic changes, etc.).

## 3. Nanoparticles for Targeting Physiological Conditions in the Tumor Microenvironment

### 3.1. Nanoparticles and Penetration into the Tumor Microenvironment

#### 3.1.1. The Enhanced Permeability and Retention Effect

The enhanced permeability and retention (EPR) effect is among the most significant consequences of tumor angiogenesis. Solid tumors depend on fast angiogenesis to conserve adequate nutrients and oxygen. The rapid increase in the number of endothelial cells in angiogenesis leads to a reduction in the density of endothelial cells, damage of tight junctions, and generation of large gaps among the cells. Therefore, tumor blood vessels do not have enough smooth muscle cell layers and pericytes, causing more fragility against the high interstitial burden and fast-changing blood flow [16,17].

Nanoparticle therapeutics directly delivered into the bloodstream must extravasate through the vascular burdens into the targeted area and release the load. Unlike small molecules, nanoparticles are unable to pass tight junctions among endothelial cells due to their rather big dimensions. On the other hand, the vessels of the tumor part have leaking structures, which permit nanoparticles of certain sizes to cross through them [18]. Since the lymphatic structure in a tumor is highly dysfunctional, the drainage leads to the aggregation of nanoparticles in the tumor site. This situation is known as the EPR effect of nanoparticles, which is the origin of passive targeting [19].

Due to the EPR effect, nanoparticles and macromolecular drugs can aim tumors more proficiently than small molecule drugs. Over the former decades, taking advantage of the high penetrability of tumor tissues has become a significant approach for the formulation and development of novel nanoparticle therapeutics for cancer therapy.

Based on the tumor type, the pores in the tumor vasculature are usually in the dimension range of 100–800 nm [16]. Particles smaller than this size can extravasate from the bloodstream into the tumor microenvironment. After extravasation, the dispersion of the nanoparticles into the tumor tissues is based on diffusion, which is reversely associated with particle size. Reversely, the nanoparticles that penetrated the tumor microenvironment can also go back into the blood circulation via the gaps in vascular structures and then are omitted by the MPS or kidney clearance. Generally, nanoparticles with sizes of 30–200 nm display better maintenance in the tissue, resulting in higher accumulation, optimum for passive targeting of the majority types of solid tumors by benefitting from the EPR effect [20].

Additionally, the shape of the nanoparticles also plays a role in EPR-based targeting [21]. Experimental and simulation findings have indicated that spherically shaped nanoparticles are more likely to have a laminar flow arrangement so that only those particles traveling close to the superficial part of the vascular structure will be capable of extravasating into the tumor. On the contrary, bar-shaped nanoparticles are not stable hydrodynamically and do not have the same flow pattern as they move in the bloodstream. The optimum hydrodynamic characteristics provide more prospects to adjust the geometrical features of nanoparticles and consequently increase their chance to pass the gaps on the vascular wall [22,23].

Based on the mentioned studies, controlling the size and shape of nanoparticles can have a significant effect on their accumulation at the tumor site, resulting in better delivery systems.

However, there are some significant drawbacks to taking advantage of the EPR effect when it comes to clinical studies. The EPR effect varies significantly between lab animals and humans. Human tumors are profoundly different from animal tumors due to tumor size, heterogeneity of the TME, and the rate of progression of the tumor. Therefore, the preclinical results obtained from animal models are usually contradictory with clinical observations [24,25].

Moreover, diverse tumoral structure, diffusion properties, and tumor vasculature in every single patient lead to different tumor accumulation in different individuals. Therefore, research is moving towards personalized approaches for individual therapy, considering all the involved parameters in the therapy process and selecting the most responsive individuals from clinical trials [26,27].

#### 3.1.2. PEGylation of Nanoparticles

Finding any strategy for increasing the hydrophilicity of the nanoparticles would result in more stable particles with increased circulation time in the body. Polyethylene glycol (PEG) is the most widely used polymer for enhancing hydrophilicity, masking the surface of NPs, and protecting them from opsonization and phagocytosis [28].

Non-specific adsorption of plasma proteins is a significant factor for the half-life of the particle in the body [29]. The resistance to protein adsorption can reduce the uptake and elimination of nanoparticles by the mononuclear phagocytic system (MPS) and increase the nanoparticle half-life [30]. The zeta potential on the surface of nanoparticles is a very important parameter for the adsorption of plasma proteins. PEGylation of the nanoparticles will result in a nearly neutral zeta potential due to the electrical neutrality of the PEG, and this will improve the anti-protein adsorption properties of nanoparticles [31].

PEG has the exceptional feature of being soluble in both aqueous and organic phases at any molecular weight. Consequently, it can provide activated functional groups at one or both ends of many functionalities. The selection of functional groups relies on whether they can react with the hydroxyl groups of the PEG. By changing a hydroxyl group at one end of the PEG, it can be linked to diverse macromolecules, drugs, liposomes, peptides, etc. Nevertheless, the use of heterobifunctional PEGs is limited because of the formation of diols, particularly for PEGs with high molecular weights [32].

Even though the PEG extends the NP’s circulation time in the blood, it can also hinder the load release or cover the functional groups of NPs at the target location. To conquer these obstacles, stimuli-sensitive PEGs can be used, which could ease cargo release. Stimuli-sensitive PEGs can respond to environmental properties such as pH and temperature and release cargo in response to these conditions [33]. Moreover, in many cases, PEGs can also act as linkers to accelerate the functionalization of nanoparticles with specific ligands of the desired targets [34].

PEGylation is also beneficial for improving the extravasation of nanoparticles inside tumors by making them more flexible and “softer” particles. Various PEG-modified nanoparticles are developed for imaging as well as therapeutic applications [28]. PEGs on the surface of NPs also contribute to colloidal stability due to steric repulsion. The steric hindrance by the PEG layer prevents particle aggregation; also, due to less serum protein adsorption, the PEGylated nanoparticles have more stability and controlled size in blood circulation. The thickness of the PEG layer and the coverage degree of the surface both depend on the molecular weight of PEG. Therefore, steric repulsion is more often observed in PEGs with molecular weights higher than 20–30 KDa [35,36,37].

The increase in MW and density of PEGs leads to a thicker PEG layer and, therefore, more neutral zeta potential values. More neutral zeta potential values result in less nonspecific adsorption of plasma proteins, therefore prolonging the blood circulation half-life of the nanoparticles [31,38,39].

The molecular weight, chain branching, length, density, and shape of the PEGs are also important factors in controlling the NPs’ surface hydrophilicity, their uptake, and their metabolic pathways in biological structures [40].

The impact of PEGylation on nanoparticles and its challenges, development of PEGylated formulations, diverse parameters in the nanoparticle preparation, their influence on pharmacokinetics and biodistribution of tumor targeting nano-systems, and the effect of the TME on internalization of PEGylated NP formulations have been extensively reviewed by Mozar et al. [41].

### 3.2. Nanoparticles Targeting the Acidic Tumor Microenvironment

In comparison with normal cells (pH 7.4), the pH value of the extracellular part of tumor cells is lower (pH 6.0–7.2). Inside tumor cells, the pH of lysosomes and endosomes is much lower (almost pH 4–6) [42]. The pH value of the extracellular section in normal tissues is ~7.4, whereas it is much lower in the TME (~6.7–10^7.1^) [43].

Numerous mechanisms are responsible for the acidic pH in tumors. First, tumor cells take advantage of aerobic glycolysis as the main metabolism pathway in the hypoxic situation, leading to greater production of lactic acid and hemoglobin (Hþ), which will later be released in the TME through active membrane-based ion transport and passive diffusion. Furthermore, the carbonic anhydrase 9 (CA9), which is overexpressed in various cancer types such as breast and lung cancer, is also responsible for the low pH conditions in the TME [44].

Moreover, various other mechanisms, such as the adjustment with hypoxia, abandoned cell proliferation, oncogene activation, and defects in tumor perfusion because of the disordered vascular structures, can also relate to the tumor acidic microenvironment [45].

Tumor cells can also impose oxidative stress on their nearby stromal cells, such as CAFs and TAMs, through the production of reactive oxygen species (ROS). This results in mitochondrial defects in TAMs and CAFs, leading to high concentrations of lactate in TME [46].

The abnormal pH in the TME leads to tumor development, metastasis, invasion, and chemoresistance and, consequently, more aggressive and fatal cancers. Thus, targeting an acidic TME is an appropriate tumor therapeutic approach [43,47].

An acidic TME provides an attractive strategy for researchers to produce several types of pH-responsive nanoparticles [48]. The pH-sensitive nanoparticles can keep their structure in blood flow and prevent the intoxication of normal tissues due to the premature release of drugs. After reaching the tumor site, they can increase the intake of drugs by the tumor cells and speed up the drug release at the tumor environment over numerous mechanisms (PEG disconnection, hydrolysis of acid-sensitive bonds, and protonation of acid-labile groups, etc.) to expand the anticancer efficiency [49]. Recently, several forms of pH-sensitive nano-sized drug delivery systems have been designed, including pH-sensitive micelles, liposomes, and hydrogels.

There are several approaches for producing pH-sensitive nanomaterial. One method is to present “ionizable” chemical groups, for instance, phosphoric acids, amines, carboxylic acids, etc., to nanoparticles. These groups, with diverse pKa values and chemical structures, can receive or donate protons and go through pH-dependent alterations in physical or chemical features, for instance swelling extent or solubility, leading to drug release. Another way is to apply acid-sensitive chemical bonds either for direct covalent binding of drug molecules to the surface of nano-systems or to build new nanoparticles. These acid-sensitive chemical bonds can maintain neutral pH values but will be degraded in an acidic environment. This specific feature makes them favorable systems for producing pH-sensitive delivery systems. The acid-labile bonds most frequently used in studies are orthoester, acetal, imine, hydrazine, and cis-aconyl linkers [50].

Among the most frequently used pH-sensitive ionizable polymers are anionic polymers consisting of carboxylic groups, for instance, poly methacrylic acid (PMAA), poly ethacrylic acid (PEAA), poly propylacrylic acid (PPAA), poly(2-n-butylacrylic acid) (PBAA), poly(N-isopropylacrylamide) (NIPAM), and poly(glycolic acid) (PGA). In an acidic environment, these polymers are protonated, and their backbones become moderately hydrophobic; on the contrary, at neutral or high pH values, they are deprotonated and become hydrophilic.

Kim et al. [51] conducted research on polymeric micelles prepared with PMAA attached to poly(ethylene oxide) (PEO) with cross-linked polyanion and pH-sensitive features for the delivery of the cationic drug doxorubicin. Doxorubicin (DOX) is a weak base and is positively charged in a physiological state. The sugar unit of DOX contains amine. However, the formulation of the micelles in the lower pH resulted in the formation of ammonium groups. In these complexes, the ammonium group in the daunosamine section of DOX electrostatically binds to the carboxylic group of the PMAA part of the PEO–PMA copolymer. The hydrophobic interactions among the anthracycline remainders of DOX are responsible for the further maintenance of the complex. At inferior pH values, protonation of carboxylic groups in the micelle cores (at acidic conditions) leads to enhanced DOX release. DOX was released at pH 5.5 from the micelles considerably quicker than at pH 7.4. At pH 5.5 up to 50% of DOX loaded into the micelles was liberated during the first hour. Nevertheless, no burst release of DOX at pH 7.4 was observed. The DOX-loaded cross-linked micelles displayed pH-sensitive drug release features.

Cationic polymers having tertiary amine, imidazole, or pyridine groups go through a pH-dependent phase conversion, leading to the release of drugs. For instance, poly beta-amino ester (PbAE) is synthesized utilizing bis (secondary amines) or primary amines and bis(acrylate ester) groups. This class of polymers goes through a hydrophobic–hydrophilic phase shift by the pH value declines from basic to acidic and is capable of solubilizing fast at pH values lower than its pKa. Consequently, in the acidic pH of the tumor microenvironment, PbAE-based nano-systems can dissolve quickly and release their cargo. Much research has confirmed the biodegradation, high drug and gene delivery efficacy, non-cytotoxicity, pH sensitivity, and tumor-inhibitory characteristics of PbAE nanoparticles [52,53].

pH-sensitive polymers with acid-responsive chemical linkers are formed to maintain stability at physiological conditions and to quickly degrade in the slightly acidic pH values of endosomes, lysosomes, and tumor tissues, resulting in fast drug release. For instance, the hydrazone bond is a pH-sensitive bond extensively used in drug delivery systems (DDS). Multi-block polyurethanes containing hydrazone bonds are designed to produce smart nanoparticles that go through gradual biodegradation. These polymers use pH-responsive PCL-hydrazone-PEG-hydrazone-PCL macrodiol (PCLH) and PCL as the soft section, L-lysine derivative tripeptide, L-lysine ethyl ester diisocyanate (LDI), and 1,4-butanediol (BDO) as the hard part, and hydrazone-linked methoxyl-PEG (mPEG-Hyd) as the ending section [54].

With this copolymer, Ding et al. have produced a pH-responsive DDS for the delivery of paclitaxel (PTX) to tumor cells. This nano-system displayed unique features, such as pH-reliant shell detachment when reaching the tumor site and intracellular drug release because of the acidity inside tumor cells. Consequently, these nanocarriers could considerably increase the biodistribution and efficacy of chemotherapeutic drugs in cancer therapy [55,56].

Zhang et al. [57] designed polyamide-amine (PAMAM) dendrimer grafted persistent luminescence nanoparticles (PLNPs) encapsulating DOX and modified with aptamer AS1411 and studied the anticancer efficiency of these particles. The results demonstrated a higher detection of luminescence signals from the PLNPs-PAMAM-AS1411/DOX in the HeLa cells compared with normal cells due to the higher uptake. Animal studies demonstrated luminescence signal detection inside the tumor tissues of the mice who received PLNPs-PAMAM-AS1411/DOX, whereas the PLNPs-PAMAM-DOX group did not show signals due to the modification of nanoparticles with the AS1411 aptamer and active targeting effects resulting in the accumulation of the nanoparticles in the tumor area.

Domiński et al. [58] designed a polymeric nanoparticle system consisting of triblock copolymer poly(ethylene glycol)-b-polycarbonate-b-oligo([R]-3-hydroxybutyrate) (PEG-PKPC-oPHB) and with DOX and 8-hydroxyquinoline glucose (8HQ-glu)- and galactose conjugates (8HQ-gal) as the encapsulated cargo. It was observed that this system could release drugs due to the increase in the hydrophilicity of the initially hydrophobic core by hydrolysis of the ketal groups in acidic pH. In vitro tests demonstrated a significant enhancement in drug release at a lower pH for this system (46% at pH 7.4 vs. 77% at pH 5.5).

Huang et al. [59] reported the production of nano-systems, which take advantage of a dual method, i.e., acidic pH values and higher amounts of matrix metalloproteinase 2 in the TME. The authors modified the surface of nanocarriers in a way that penetration of the whole system is eased by a cover of a pH-responsive peptide (secondary cover) on a stimulated cell-penetrating peptide (primary cover). Further tailoring is through linking the matrix metalloproteinase-2 (MMP2) substrate. When this nano-system arrives in the TME, the initial attraction of the nano-system to the moiety will reduce due to the acidic pH, and simultaneously, the MMP2 substrate will be cut, permitting the stimulated cell-penetrating peptide for enhanced penetration into tumor cells. The researchers studied the encapsulation of plasmid DNA, cellular uptake, tumor-targeting efficacy, toxicity, and gene transfection and confirmed the nanoparticle to have considerable efficiency in cancer-targeting.

Li et al. [60] formulated a mannose-doping doxorubicin-loaded mesoporous silica nano-system (MSN-Man-DOX) covered by polydopamine-Gd3+ (PDA–Gd) metal-phenolic networks. This nano-system was also adjusted by poly (2-Ethyl-2-Oxazoline) (PEOz), resulting in a novel nano-system MSN-Man-DOX@PDA-Gd-PEOz. Various properties of this system, such as pH-responsiveness due to charge reversal, drug release, biodegradation, and magnetic resonance imaging (MRI) potential were studied in vitro. The results demonstrated significant tumor penetration features of this nano-system, with promising treatment efficiency with the synergistic effect of chemotherapy and photothermal therapy (PTT). The involvement of a magnetic resonance imaging contrast agent would aid the monitoring properties of this system, leading to an efficient “theranostic” nanoparticle.

In another study, Son et al. designed polymeric micelles using poly (ethylene glycol-block-cyclohexyloxyethyl glycidyl ether)s (mPEG-b-PCHGE) with an acetal group as the pH-sensitive linkage. Increased stability and better pH responsiveness were observed as a result of the addition of the hydrophobic CHGE block [61].

In another approach, PEG-coated nanoparticles, which are stable in blood and normal physiological conditions but can be degraded in the TME, can achieve a depegylation-based targeted delivery in the TME and intracellular drug delivery leading to more efficient drug delivery and decreased side effects [62].

As an example, in a recent study Dominski et al. [63] synthesized a diblock copolymer, a poly(ethylene glycol)-hydrazone linkage-poly[R,S]-3-hydroxybutyrate, capable of self-assembling into micelles with nano-dimensions. The polymer contains a hydrazon bond between the two copolymer blocks that, under an acidic pH, causes the shell-shedding of micelles leading to drug release (8-hydroxyquinoline glucose, galactose conjugates as and doxorubicin). Based on DLS studies, the micelles demonstrated stable behavior at pH 7.4 but degraded at acidic pH. Additionally, in vitro apoptosis, cytotoxicity, and life cycle assays demonstrated that the drug-loaded micelles were highly effective against diverse cancer cells.

Wang et al. [64] designed a PEG-modified liposome system encapsulating irinotecan, which is able to expose the ligand by cleavage of the bond in reduced pH conditions. The system was able to hide the ligand in its outer layer while it was able to expose them in reduced pH conditions, such as tumor microenvironment.

Liu et al. [65] formulated hollow Fe–GA nanospheres by implementing bovine serum albumin (BSA) encapsulating DOX for MRI-guided synergistic chemotherapy/CDT/PTT. This pH-sensitive hollow Fe–GA/BSA@DOX system was able to be degraded in the TME and release loaded DOX, along with Fe(III) ions. Furthermore, Fe(III) could reduce the overexpressed GSH in cancer cells to form Fe(II) to enable the Fenton reaction. On the other hand, the system was able to rapidly convert NIR light into heat. In vitro and in vivo MRI studies also demonstrated the improved imaging performances of Fe–GA/BSA, confirming this system as a reliable platform for synergistic cancer MRI and chemotherapy, CDT, and PTT.

Du et al. [66] produced a nanogel modified with amino groups with a negative-to-positive conversion characteristic. This nanogel remains intact at neutral and high pH values, but in acidic pH, the amino groups are cleaved, and the positive charge of the nanogel promotes cellular uptake.

### 3.3. Nanoparticles Targeting the Hypoxic Tumor Microenvironment

Hypoxia is one of the significant characteristics of the TME. The rapid replication of cancer cells hastens the intake of oxygen and, as a consequence, lowers the oxygen level in solid tumor zones [67].

As tumors develop, their oxygen requirement increases, which promotes the nearby cells to release several growth factors involved in the process of angiogenesis (growth of new blood vessels). However, the structure of these new blood vessels is immature and weak, resulting in leakage and poor perfusion [68]. Hypoxia also contributes to up-regulating the expression of chemoattractant chemokines ligand (CCL) 22 and 28 and the accumulation of Treg and myeloid-derived suppressor cells (MDSCs) [69,70]. Hypoxia also increases the expression of T cell immunoglobulin domain and mucin domain-3 (TIM-3) and CTLA4 on Tregs and tumor-associated macrophages (TAMs), leading to immunosuppression [71,72]. Furthermore, hypoxia induces therapeutic resistance to other cancer therapy approaches where the presence of oxygen molecules is fundamental for cancer cell elimination, such as photodynamic therapy and radiotherapy [73]. Hypoxic zones of tumors are less disposed to chemotherapy because of the restricted drug delivery by the bloodstream. In the hypoxic area, tumor cells can apply many methods to survive, for example, erythropoietin (EPO) production, changing from aerobic to anaerobic metabolism pathway, relaxing DNA repair systems, employing the stromal components, along with upregulating hypoxia-inducible factor (HIF) 1a and HIF 2a [74,75]. Hypoxia has a connection with genotype selection too, which is desirable for tumor development since tumor protein 53 (TP53) mutations support cell survival in damage happening because of hypoxia and re-oxygenation, resulting in an anabolic shift in principal metabolism pathways. This situation of low oxygen levels stimulates tyrosine-kinase-mediated signaling pathways and leads to angiogenesis, invasion, and metastasis, which are also boosted because of the start of the epithelial-mesenchymal transition of cells (EMT). Along with all the mentioned effects, hypoxia is reported to interrupt DNA repair pathways and result in genomic instability due to the enhanced formation of reactive oxygen species [76].

There are two approaches for targeting the hypoxic area, first, designing drug delivery systems (DDSs) for releasing therapeutic agents in the hypoxic environment, and second, designing DDSs for modifying the hypoxic environment.

Designing a DDS that releases the cargo drug in the hypoxic microenvironment is an important method for benefitting from the hypoxic TME [77].

Huo et al. designed a size-tunable nanosized bomb with a primary size of almost 33 nm, presenting an extended half-life during circulation and degraded for releasing small hypoxia microenvironment-targeting nano-systems to obtain increased tumor penetration. Since CCL-28 chemokine is overexpressed in the hypoxic conditions of the TME, the nanoparticles were modified with ligands of this chemokine, and then the clusters of these nanoparticles were covalently bound with a matrix metalloproteinase cleavable peptide [78].

Thambi et al. produced a hypoxia-sensitive polymeric nano-system. A hydrophobically adjusted 2-nitroimidazole derivative was linked to the carboxymethyl dextran backbone (CM-Dex). Doxorubicin (DOX), as a model drug, was successfully encapsulated in the hypoxia-responsive nanoparticles (HR-NPs). It was observed that nanoparticles released DOX at a sustained speed in physiological conditions, but the release profile had a dramatic increase in hypoxic conditions. These nanoparticles demonstrated efficacy in both in vivo and in vitro tests [79].

**Figure 2:**
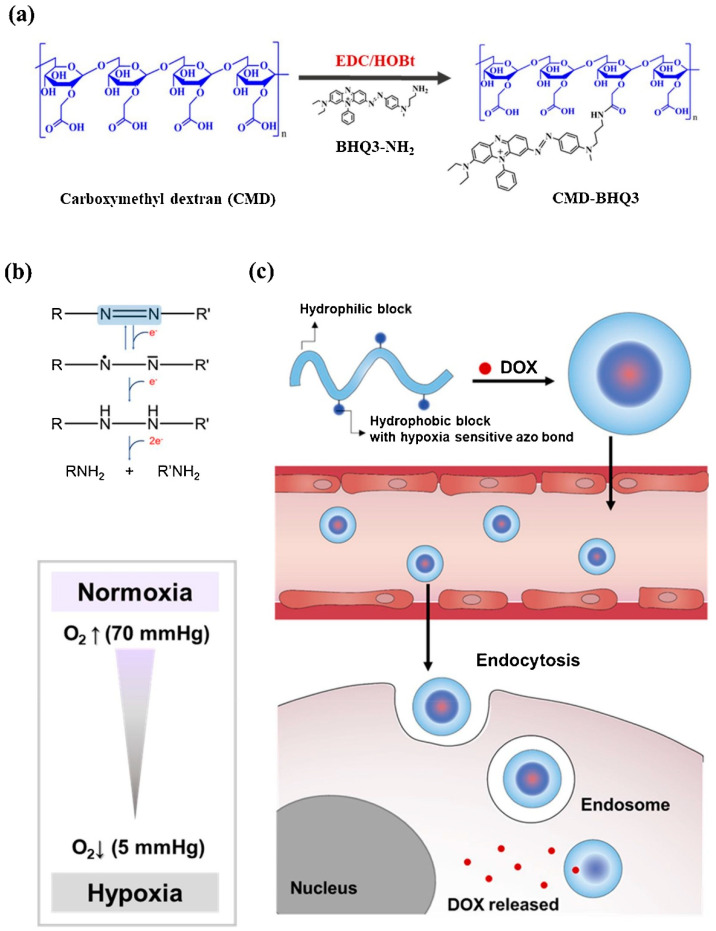
(**a**) Schematic formation of the hypoxia-sensitive amphiphilic polymer complex. (**b**) Reduction of azobenzene derivatives. (**c**) Schematic demonstration of the DOX-loaded CMD-BHQ3 NPs (DOX@CMD-BHQ3 NPs) and their hypoxia-responsive release behavior. DOX@CMD-BHQ3 NPs accumulate in the tumor environment due to the EPR effect, and the drug release occurs under hypoxic conditions of the intracellular environment. Reprinted from [80].

Son et al. designed a hypoxia-sensitive nanoparticle, including black hole quencher 3 (BHQ3) and carboxymethyl dextran (CMD). The amphiphilic polymer complex undergoes self-organization in the water environment and forms CMD-BHQ3 NPs. The anticancer agent, doxorubicin (DOX), was efficiently loaded in CMD-BHQ3 nano-systems to form DOX@CMD-BHQ3 nanoparticles. It was reported that the release degree of DOX improved noticeably under hypoxia conditions by means of breakage of the azo bond in BHQ3 [80]. Figure 2 depicts the chemical structure and formulation of the system developed in this study and its drug-release behavior under hypoxic conditions.

Thambi et al. designed a novel system of hypoxia-responsive polymeric micelles (HS-PMs) by the usage of poly(ε-(4-nitro)benzyloxycarbonyl-L-lysine) as the hydrophobic block and poly(ethylene glycol) as the hydrophilic block. Because of its amphiphilic nature, the block copolymer-shaped micelles and doxorubicin were encapsulated in the micelles in an aqueous solution [81].

As mentioned before, modifying the hypoxic environment of the TME is also another method for targeting hypoxic conditions in the TME.

Liu et al. reported increased chemo-responsivity in leukemia cells (K562) under hypoxic conditions using polyethylene glycol-poly l-lysine-polylactic-co-glycolic acid-based nano-systems modified with transferrin and loaded with model drug daunorubicin. Nanoparticles were reported to have a down-regulation influence on HIF-1α and stimulated apoptosis to conquer hypoxia [82].

Zhu et al. produced a nano-system containing a functional shell consisting of 2-deoxyglucose (DG)-polyethylene glycol (PEG) linked with the complex of lipoic acid, lysine, and 9-poly-d-arginine (LA-Lys-9R) by means of a hydrazone bond. The core of the system consists of CdTe quantum dots (QDs). This system displayed higher antitumor effects and lower toxicity by effective targeted delivery of HIF-1α siRNA to hypoxic tumor cells [83].

Abbasi et al. reported the production of clinically suitable preparations of mixed manganese dioxide (MnO_2_) nanoparticles (MDNP) by consuming biocompatible materials for re-oxygenating the TME by interactions with endogenous H_2_O_2_. Boosted efficiency of radiation therapy applied after the consumption of these nano-systems was observed in extremely hypoxic murine or human xenograft breast cancer models [84].

Nanoparticles can also contribute to enhancing the efficacy of other cancer therapies (such as radiotherapy and photodynamic therapy), but their effect is limited due to hypoxia in the tumor site [85,86].

Gao et al. [87] developed a red blood cell (RBC) membrane-coated PLGA nanoparticle encapsulating perfluorocarbon (PFC). PFC has high oxygen solubility and can be loaded with high amounts of oxygen. This nanoparticle demonstrated high oxygen binding and an extended retention time. Due to its very small size, this nanoparticle easily leaks from blood vessels and releases its cargo (oxygen) in the TME by diffusion [88].

Song et al. [89] developed polyethylene glycol stabilized nanodroplets modified with tantalum oxide (TaOx) nanoparticles as a radiotherapy sensitizer system. In this system, PFC can steadily release oxygen, and TaOx nanoparticles can absorb X-rays to eliminate cancer cells. This dual approach can increase the efficacy of radiotherapy as well as oxygenate the tumor environment. It was observed that tumor oxygenation had a three-fold increase after injecting this nano-system and hypoxic areas shrank significantly.

In another study, Song et al. [90] developed a bismuth selenide (Bi_2_Se_3_) nanoparticle encapsulating PFC. Bismuth selenide is a photothermal agent, which releases heat when exposed to a near-infrared laser, and this heat induces the release of oxygen from PFC, which could be used as a tumor oxygenation strategy.

### 3.4. Nanoparticles Targeting the Reductive Tumor Microenvironment

It has been proved that the tumor microenvironment has reductive properties due to high concentrations of reactive oxygen species (ROS) and reduced glutathione (GSH). These agents are usually present in organisms in normal conditions. However, their expression undergoes abnormalities under pathological circumstances, such as cancer and inflammation [91,92].

ROS refers to a huge variety of intermediate species with or without oxygen atoms, either in the form of radical or non-radical. Radical species include (O_2_^•−^), hydroxyl radical (HO^•^), alkoxyl radicals (RO^•^), peroxyl radicals (ROO^•^), thiyl peroxyl radicals (RSOO^•^), sulfonyl radicals (ROS^•^), and thiyl radicals (RS^•^). Whereas non-radical molecules include hydrogen peroxide (H_2_O_2_), singlet oxygen (O_2_), and ozone/trioxygen (O_3_). Other types of reactive species contain nitrogen atoms, such as nitric oxide (NO^•^), peroxinitrite (ONOO–), nitrocarbonate anion (O_2_NOCO_2_–), nitrosoperoxycarbonate anion (O = NOOCO2–), dinitrogen dioxide (N_2_O_2_), peroxynitrite (ONO), etc. Inflammatory cells, particularly macrophages and neutrophils, encourage producing ROS during immune or inflammatory reactions [93,94].

It has been proven that ROS levels have significant roles in regulating the signaling pathways associated with cell growth, survival, and replication at physiological conditions. Diverse cells, such as CAFs, ECs, and inflammatory cells, as well as pathological conditions, such as angiogenesis and hypoxia, contribute to ROS generation [95].

Additionally, higher levels of ROS are connected to a higher level of antioxidants [96]. Intracellular GSH acts as a powerful natural antioxidant. As a result, levels of both ROS and GSH are increased in the tumor microenvironment. Elevated levels of intracellular GSH save cells from cell death and apoptosis and induces the survival of tumor cells. The increased level of GSH is also related to the resistance to chemotherapy [97].

GSH is a significant enzymatic system that keeps the intracellular redox balance in tumor cells. Increased levels of GSH are associated with elevated levels of GSH-related enzymes such as γ-glutamyl-transpeptidase (GGT) and γ-glutamylcysteine ligase (GCL) activities along with a higher expression of GSH-transporting pumps. GSH is a tripeptide consisting of cysteine, glutamic acid, and glycine that is in two forms: reduced form (GSH) with a millimolar concentration in the intracellular section, and oxidized form (GSSG), which forms less than 1% of the total GSH in the body. Mitochondria contain almost 10% of GSH, and about 90% of that is found in the cytosol. The main duty of GSH is the detoxification of endogenous compounds and xenobiotics and the preservation of intracellular redox balance [98]. Moreover, the H_2_O_2_ concentration in cancer tissue is ≈1000 times more than that of healthy tissue (20 × 10^−9^ m) [99].

Pharmaceutical technology can take advantage of the redox condition of the TME for the synthesis of nanoparticles that can degrade and release their cargo, specifically in reductive areas. Different types of redox-sensitive nanoparticles have been designed and studied in the past few years, such as diverse polymeric nano-systems, mesoporous silica nanoparticles (MSNs), nanogels, and nanoliposomes. Redox-sensitive chemical groups can be applied in the structure of nanoparticles either as a linker between the particle and cargo or as a part of the nanoparticle’s chemical structure.

ROS-responsive bonds include sulfur-containing, selenium (Se)-containing, tellurium (Te)-containing, and arylboronic ester linkages. Some of the most popular groups applied for linkages are arylboronic esters, disulfide, sulfhydryl, thioketal, thioeter, diselenide, and peroxalate ester [100,101].

Yin et al. modified the surface of liposomes containing (DOX) with chitooligosaccharides (COS) by a disulfide linkage, which remained intact under physiological conditions and was capable of penetrating into the tumor cells due to the adhesive characteristics of COS. After entering tumor cells, intracellular GSH helps the breakage of the disulfide bond and releases the cargo into the cytosol [102].

To increase the targeting delivery, modified liposomes adjusted with reduction-sensitive conjugates have been produced. For instance, Yin et al. made modified cationic liposomal nanoparticles via chotooligosaccharides (COS, MW2–5 KDa) as the reduction-responsive link and attached estrogen on the surface of the liposomes to increase targeted delivery to osteosarcoma [103].

Kumar et al. produced folic acid and trastuzumab-modified multiblock copolymeric nanocarriers using 2-hydroxyethyl disulfide, which resulted in a high drug release profile in the presence of 10 mM GSH at pH 5.5 [104].

In another work, Conte and coworkers linked hydrophilic PEG and hydrophobic poly(lactic-co-glycolic acid) (PLGA) at both ends of the disulfide bond to obtain a novel amphiphilic diblock copolymer to increase uptake in lung cancer cells. In this research, non-redox-responsive NPs (nRR-NPs) and redox-responsive NPs (RR-NPs) were obtained by PLGA-PEG and PLGA-S-S-PEG, respectively. Comparative tests between nRRNPs and RR-NPs displayed that RR-NPs had increased uptake and faster degradation in lung cancer models as a result of the decomposition of the disulfide bonds by GSH [105].

Wu et al. [106] constructed a GSH, pH, and NIR light triple-sensitive nano-system based on magnetic hollow and porous carbon nanoparticles (MHPCNs) for synergistic photothermal/chemotherapy guided by MRI. In this platform, SiO_2_ was used for absorbing ferrocene, and H_2_O_2_ treatment was used afterward to form SiO_2_@Fe_3_O_4_@C NPs. After the elimination of the SiO_2_ core, the carboxylic groups of MHPCNs were covalently bonded to cystamine dihydrochloride to form MHPCNs–SS. Afterward, pH-sensitive poly(γ-glutamic acid) (PGA) was used to block the pores with DOX-loaded particles. In addition, the particles were modified by folic acid (FA) to improve the targeting ability of MHPCNs– SS–PGA/DOX. When the MHPCNs–SS–PGA–FA/DOX system enters cancer cells via endocytosis, the PGA layer is separated due to the low pH and high GSH level, leading to the drug (DOX) release. In addition, the release of DOX could be increased by laser irradiation. These particles demonstrated improved tumor suppression with decreased side effects.

Redox-responsive nanogels are also used for targeted tumor delivery and adjustable drug release. In the synthesis process, polymers and biomaterials containing disulfide bonds are used for the production of nanogels sensitive to the intracellular reductive environment. A vast range of polymers such as polyvinyl alcohol (PVA), alginate (AG), and poly(ethylenimine) (PEI) have been applied in reduction-sensitive nanogel structures with disulfide bonds [107,108].

Deng et al. reported a thiolated-hyaluronic-acid-based redox-sensitive nanohydrogel system used for the encapsulation of oncolytic viruses. It was observed that the nanohydrogels were degraded within 10 h in the reductive environment due to the breakage of disulfide bonds, but they remained stable in normal physiological conditions for up to 5 days [109].

In another work, Deng et al. produced a novel core-shell structure nanoparticle system consisting of vinyl sulfonated poly(N-(2-hydroxypropyl) methacrylamide mono/dilactate)-polyethylene glycol-poly(N-(2-hydroxypropyl) methacrylamide mono/dilactate) triblock copolymer in the shell and thiolated hyaluronic acid in the core. This nano-system also displayed high internalization efficiency redox responsive protein release into macrophages [110].

### 3.5. Nanoparticles Targeting the Metabolic Changes in the Tumor Microenvironment

There are various alterations and shifts in the metabolic pathways of tumor cells. Due to their high replication rates, cancer cells mostly have a high metabolism. The most studied metabolic pathway is the change in aerobic glycolysis in cancer cells, also known as the Warburg effect. This phenomenon is described by the tendency of highly proliferating cells (including cancer cells) to consume glucose and release the carbon as lactate even in the presence of oxygen [111,112]. Highly proliferating cells have the propensity for expressing glucose transporters and glycolytic enzymes. [113]. Tumor cells significantly rely on aerobic glycolysis to obtain energy instead of the classical pathway of mitochondrial oxidative phosphorylation (OXPHOS). The Warburg effect was first considered one of the fundamental “causes of cancer”; however, further studies indicated that tumor suppressor genes and mutations of oncogenes might be the main reason for malignant transformation and aerobic glycolysis is rather an epiphenomenon than a cause [114,115].

Other different nutrients also contribute to the metabolic functions of different cancer cells. Recent research revealed a various number of fuels, such as branched-chain amino acids, serine, lactate, and glycine, participating in metabolic pathways for different tumors’ progression. More importantly, there are other metabolic changes corresponding to new functions of tumor cells, such as changes in fatty acid beta-oxidation, TCA cycle, and anabolic metabolism [116]. However, glutamine metabolism is reported as one of the most common pathways in different cancers [117].

Elgogary et al. studied nano-systems of bis-2-(5-phenylacetamide-1,2,4-thiadiazol-2-yl) ethyl sulfide (BPTES) glutaminase inhibitor. These nanoparticles were formed to display effects on the glutamine metabolism pathway. Results approved that these nanoparticles reduce tumor growth by mainly targeting replicating cancer cells resulting in a promising method for pancreatic cancer therapy [118].

Moreover, in the last few years, 3-bromopyruvate (3-BrPA), which is a small molecule agent and a very effective glycolytic inhibitor, has shown promising results as an anti-tumor drug. This drug has the capability to inhibit numerous glycolytic enzymes [119,120,121].

Recently, there has been a lot of research on better formulating this drug. Several studies applied liposomal nanoparticles for specific delivery of the 3-BrPA to tumor sites. 3-BP is taken up in tumor cells via monocarboxylate transporter 1 (MCT1), which is overexpressed in a variety of tumor cells, especially in hypoxic tumor areas [122].

For further developing the targeting, peptides or proteins targeting tumor-specific receptors were linked to the surface of the nanoparticles, and it was observed that the liposomal formulations selectively targeted tumors or spheroids and had significant effects in comparison with the unformulated 3-BrPA.

Gandham et al. developed liposomal nanoparticles with a lipid bilayer consisting of 1,2-distearoyl-sn-glycero3-phosphocholine (DSPC), cholesterol, and 1,3 distearoyl-snglycero-3-phosphoethanolamine-N-[methoxy(polyethylene glycol)-2000] (DSPE-MPEG-2000), which could encapsulate 3-BPA in its hydrophilic core. For further targeting efficacy, the surface of liposomes was modified with an EGFR-specific peptide known as GE11 (YHWYGYTPQNVI), which can be coupled to the liposome surface via a heterobifunctional PEG chain with maleimide (MAL) functionality. The efficacy of the nanoparticles was studied for penetration inside ovarian cancer spheroids, and it was observed that the liposomal nano-systems had improved cytotoxic effects in spheroids in comparison with the solution form of 3-BPA [123].

Zhang et al. encapsulated 3-BP in liposomal nanoparticles covalently functionalized with a tumor-targeting pentapeptide (CysArg-Glu-Lys-Ala; CREKA) to their surface. It was observed that nanoparticles targeted the tumors after systemic administration in tumor-bearing mice with the least side effects in major organs [124].

### 3.6. Nanoparticles Targeting the Extracellular Matrix of Tumor Microenvironment

Extracellular matrix (ECM) controls cell replication by the complex effects of its components, including proteoglycans, collagen, elastin, polysaccharides, hyaluronan, various enzymes, and growth factors. When these components assemble, they result in various biological tasks and regulation of cell activities [125]. The ECM consists of more than one hundred proteins, forming a network and acting as a platform for cell progression. The ECM also consists of numerous cytokines, growth factors, and chemokines released by tumoral and stromal cells [126].

Physical features of ECM, such as density, porosity, rigidity, and orientation, could explain the scaffolding and backup pathways of ECM in keeping the integrity of the connected tissue. In the TME, mechanical, chemical, and physical characteristics of ECM have re-formed a way that they could encourage carcinogenesis along with metastasis. Increased binding of collagen and components originating from lysyl oxidase results in higher stromal rigidity and density. The enhanced collagen crosslinking offers improved mechanical support for tumor progression and simultaneously obstructs the penetration of chemotherapeutic drugs, thus resulting in drug resistance [127].

#### 3.6.1. Collagen

Collagen is an essential element of the ECM. Several reports have revealed that collagen contributes to tumor progression. Collagen ratio and cross-linking are significantly associated with tumor stiffness. Higher stiffness is also related to the poor prognosis of various types of tumors, such as breast tumors [128,129,130]. Therefore, targeting collagen in the TME seems a promising plan to inhibit tumor initiation, growth, and progression [131].

A common approach is to co-deliver the nanoparticles with collagenase. This co-delivery could be based on a modification of the surface of nanoparticles with collagenase or encapsulation of collagenase.

Based on the first approach, Murty et al. developed PEGylated gold nanoparticles modified with collagenase and studied their behavior in a murine tumor xenograft. Within the tumor, collagenase-modified nanoparticles showed a 35% increase in accumulation in comparison with unmodified nanoparticles [132].

Villegas et al. developed a novel nano-system with collagenase encapsulated in pH-responsive polymeric nano-capsules. The polymeric shell consisted of acrylamide (AA) as a structural monomer, hydrochloride salt of 2-aminoethyl methacrylate (Am) for providing amino groups on the nano-capsule surface, and ethylene glycol dimethacrylate (EG) as pH-sensitive cross-linker. These nano-capsules were attached to the surface of mesoporous silica nanoparticles. This hybrid nanoparticle system showed a significantly high penetration into human osteosarcoma cells (HOS), which contain a high amount of collagen in the ECM and could provide a platform for the delivery of anti-cancer drugs in cancerous tissue [133].

Zinger et al. reported the design of nano-liposomes containing collagenase (collagozome) for cancer therapy of pancreatic ductal adenocarcinoma (PDAC). It was reported that PDAC tumors, undergone pretreatment with the collagozome and followed by applying paclitaxel micelles were 87% smaller in size than the tumors pretreated by blank liposomes and then treated with paclitaxel micelles and digesting the ECM did not show any increase in the quantity of circulating tumor cells in the blood or metastasis [134].

Liu et al. produced collagenase containing nano polymers based on Mn^2+^ and an acid-responsive benzoic-imine organic bond and then adjusted by polyethylene glycol (PEG). These nano-systems displayed effective aggregation inside the tumor site so that collagenase would be liberated due to the degradation of nanoparticles in the acidic TME. The released collagenase can particularly destroy collagens, the significant factor of ECM, resulting in a relaxed ECM structure, relieved hypoxia, and improved tumor perfusion. Therefore, the secondary nanoparticles, chlorin e6 (Ce6)-loaded liposomes (liposome@Ce6), would display higher penetration inside the tumor [135].

#### 3.6.2. Lysophosphatidic Acid and Matrix Metalloproteinase Protein

Lysophosphatidic acid (LPA) is a fundamental part of the TME. It is mostly produced from lysophosphatidylcholine (LPC) by the autotaxin enzyme (ATX). LPA triggers six G-protein-coupled receptors to adjust cell proliferation, survival, and relocation; LPA signaling is vital for vasculogenesis in embryonic growth [136]. It acts as a pro-oncogenic and profibrotic element and has a significant part in tumor development, angiogenesis, metastasis, and drug resistance [8,137].

Loskutov et al. prepared PLGA-PEG nano-systems for the delivery of small molecule Ki16425 (an LPA signaling inhibitor) to the brain cells. Stopping the LPA signaling with the small molecule Ki16425 in highly replicative astrocytes or patient-derived glioblastoma cells/xenografts greatly destroys their development in vitro and in vivo. Furthermore, the brain delivery of Ki16425 by PEG-PLGA nano-systems suppressed tumor development in an intracranial glioblastoma patient-derived xenograft model [138].

Matrix metalloproteinases (MMPs) are a group of zinc-reliant endopeptidase enzymes. MMPs are made as inactive zymogens, which consequently are activated by means of serine proteinases or other MMPs [139].

Since MMPs can degrade nearly all contents of the ECM, they have significant roles in ECM conversion. Several studies have shown that improved expression or/and stimulation of MMPs is responsible for oncogenesis, invasion, and metastasis [140].

Sun et al. encapsulated the chemotherapeutic agent paclitaxel into nanoparticles adjusted with an MMP-sensitive linker and a cell-permeating peptide. These modified nanoparticles displayed a high affinity to cancer cells and TAMs, and the encapsulated paclitaxel was efficiently targeted and released in the tumor site due to great levels of MMPs in the TME [141].

In another study, Anajafi et al. conjugated nuclear-localizing peptide sequences (NLS), which became activated in the presence of matrix metalloproteinase-7 (MMP-7) enzyme to the surface of redox-sensitive polymersomes for the delivery of curcumin and DOX to the pancreatic cancer cell nucleus [142].

As mentioned before, Huang et al. reported the production of nanoparticles, which are targeted by a dual method, i.e., taking advantage of acidic pH and high amounts of matrix metalloproteinase 2 in the TME [59].

**Figure 3:**
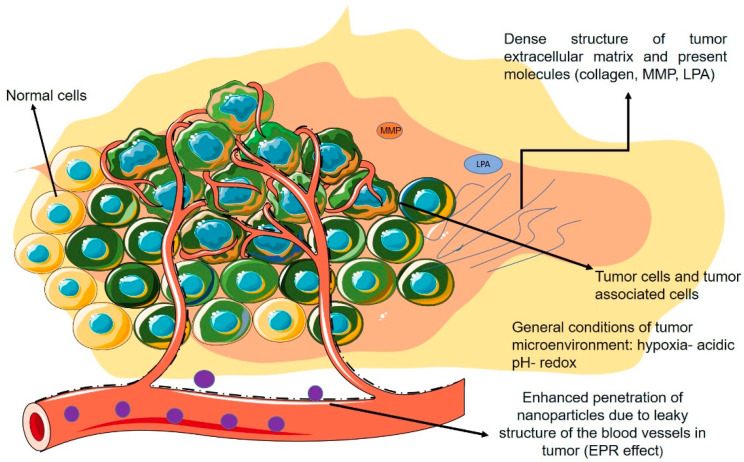
Schematic view of TME and its properties.

Figure 3 demonstrates a schematic figure of the TME and its general characteristics with a focus on the non-cellular properties mentioned in the previous section.

## 4. Nanoparticles Targeting Cells in the Tumor Microenvironment

### 4.1. Nanoparticles Targeting Tumor-Associated Immune Cells

Immune response to any type of cancer is dependent on the stage and has varied effects on the start and development of the tumor. Primarily, in the early phases of oncogenesis, cancer cells are defenseless and recognizable to immune cells, particularly CD8+ T cells and natural killer cells (NK cells), which limit tumor formation by deleting these cells. In the mid-stages of tumor progression, cancer cells choose several mechanisms for escaping the immune system. Scientists have reported a decrease in immune antigens, resulting in immune resistance. A premetastatic condition is developed by these immune intermediaries, which initiates the invasion and metastasis procedure by releasing matrix-digesting enzymes. In the last phase of cancer development, increasing cancer cells take advantage of macrophages and mast cells, which are helpful for the additional growth of the tumor via several matrix-digesting enzymes [143,144].

The role of immune cells is varied based on the different stages of cancer; they can stimulate tumor progression due to the produced TME and their connections with cellular sections of the TME. Improvements in this area consist of FDA approval for monoclonal antibodies of programmed death-1 (PD-1), cytotoxic T-lymphocyte-associated antigen-4 (CTLA-4), and programmed death-ligand-1 (PD-L1) for the treatment of small-cell lung cancer, advanced melanoma, and metastatic bladder cancer. Even though these antibodies are known to stimulate tumor T cell reaction, their non-particularity and adverse side effects as autoimmune responses are still challenges to overcome [145,146].

Chen et al. reported the production of nano-systems for primary tumor treatment resulting in the prevention of metastasis and relapses. Both indocyanine green (NIR heaters) and imiquimod (immune adjuvant) were included in PLGA nanocapsules for photothermal therapy of primary tumors accompanied by the checkpoint-blockade approach by using CTLA4. The group reported the nanoparticles stimulated the immune activity similar to vaccination in combination with a CTLA4 checkpoint-blocking agent [147].

A major ratio of the immune cells related to fast-developing tumors belongs to tumor-associated macrophages (TAMs). Macrophages are a major component of the mononuclear phagocyte system and play significant roles in the procedure, such as tissue repair and wound healing [148,149]. Macrophages undergo differentiation in response to different signals. M1 and M2 are known as the two important phenotypes of macrophages. The M1 phenotype forms after exposure to IFN-γ and lipopolysaccharides (LPS) and releases high levels of interlukin 12 (IL-12). The M2 phenotype is formed in response to IL-4 or IL-13 and secretes high levels of IL-10 [150,151,152]. These phenotypes have a dissimilar influence on tumor development after arriving at the third phase (escape). The M1 phenotype is cytotoxic and phagocytic and able to reduce tumor growth, whereas the M2 phenotype is reported to be immunosuppressive. The TME mostly stimulates and retains macrophages in the M2 phenotype by secreting several growth factors and cytokines [153,154,155]. Some of the markers overexpressed on M1 include HLA-DR, CD86, pSTAT1, and the markers on M2 phenotype include CD163, CD204, and [156]. TAMs secrete cytokines and VEGF, which have important roles in the immunosuppression and angiogenesis process, as well as tumor invasion and metastasis [153].

Formerly, an IL-4-induced M2-like murine macrophage binding peptide called M2pep was developed and displayed efficiency in reversing the M1/M2 (F4/80 + low/high) ratio in a CT26 tumor model [157]. João Conde et al. produced an M2 peptide–PEG- and siRNA-modified gold nanoparticle (M2pep-RNAiAuNP) for lung adenocarcinoma treatment. In comparison with a non-targeting peptide, M2pep-modification improved F4/80 + TAM uptake of the nano-systems injected into a lung tumor, and the particles were stable for more than 3 weeks after injection. RNAi-mediated VEGF silencing reduces TAM, decreases tumor growth, and prolongs survival [158].

Antibody-targeted nanoparticles also have been produced to increase T-cell response in the tumor microenvironment. CD8-targeted nanoparticles were synthesized by Schmid et al. using PD-1 F(ab)2 on the surface of PEG-PLGA nanoparticles encapsulating either SD-208, an inhibitor of TGFBR1, or a TLR7/8 agonist for leading the lymphocytes to tumors. The mentioned nanoparticles were commonly taken up by PD-1+ T cells in the tumor region rather than circulating T cells because of higher PD-1 expression in tumor environment T cells. It was observed that PD-1+ T cell-targeted delivery of TLR7/8 agonist (R848) increased the percentage of CD8+ T cells in the TME and improved therapeutic response to checkpoint inhibition [159].

Yang et al. designed a nanoparticle system capable of delivering small-molecule drugs. Gold nanoparticles protected with amphiphilic organic ligands have shown efficiency in targeting lymphocyte populations. The TGFBR1 inhibitor-loaded gold nanoparticle was decorated with an anti-CD8 antibody and displayed increased uptake by peripheral blood CD8+ T cells. This delivery system enhanced the therapeutic effect of anti-tumor vaccination via stimulating IFN-γ- and TNF-α-expressing cytotoxic T cells [160].

Bisphosphonates are found to be active against TAMs but also possess a short half-life as a major drawback. Their pharmacokinetic features can be modified by applying nanoparticle approaches [161].

Sharma et al. reported the formulation of PLGA nanoparticles containing clodronate (a drug from the category of bisphosphonates) with the W/O/W emulsion technique. The outcomes demonstrated that drug-loaded nano-capsules display considerable inhibitory effects on macrophage-like RAW264. Additionally, the authors modified the surface of nano-capsules with the LyP-1 peptide (TAM-targeting peptide) and reported improved accumulation of modified nanoparticles in tumor sites of the cancerous mice [162].

In another study, Zhu et al. formulated PEG-covered and -modified by mannose nano-systems as transporters of several drugs into TAMs by active targeting. The authors observed less uptake of these targeted nano-systems by ordinary macrophages, hence decreasing the toxicity and side effects of TAM-targeted drugs. The idea was effective targeting of TAMs by means of the mannose–mannose receptor interactions after the degradation of acid-sensitive PEG in the acidic TME [163].

He et al. [164] prepared a mannosylated carboxymethyl chitosan (MCMC)/hyaluronan (HA) nano-vector for delivering oligodeoxynucleotides (ODN) to target macrophages. This dual-targeting nano-vector showed better immune stimulation in comparison with mono-targeting systems containing either HA or MCMC and had an influence on MCF-7 tumor cells.

**Figure 4:**
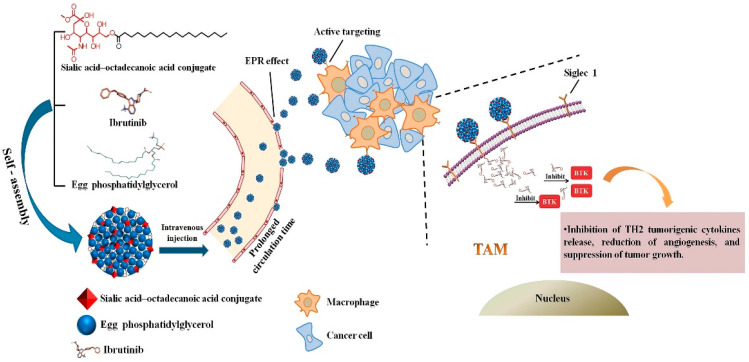
Graphic illustration of SA/IBR/EPG nano-system, tumor accumulation due to the EPR effect, and inhibition of Bruton’s tyrosine kinase function in TAMs leading to immunotherapeutic effects. Reprinted from [165].

Qiu et al. [165] developed a nanoparticle consisting of sialic acid (SA) egg phosphatidylglycerol (EPG) encapsulating Ibrutinib (IBR). IBR is an irreversible Bruton’s tyrosine kinase (BTK) inhibitor, and BTK is overexpressed in TAMs. It was observed that these nano-complexes were accumulated in TAMs and demonstrated high anti-tumor activity. Figure 4 demonstrates the chemical structure and formation process of the developed nanoparticles and their active targeting for TAMs.

There are also reports explaining the role of nanoparticles in boosting the immune response to tumors. Ordikhani et al. produced PLGA nanoparticles encapsulating anti-PD-1 antibodies to increase the antitumor efficiency and reported them to improve the effectiveness of immune checkpoint inhibitors. Furthermore, the authors highlighted the importance of secondary lymphoid tissues for moderating the related toxicities [166].

### 4.2. Nanoparticles Targeting Cancer Stem Cells

Cancer stem cells (CSCs) are stem cells known by their capacity for differentiation to form nearly all cell types existing in the tumor. By being exposed to any new system, these cells can form numerous cell types that contribute to tumor development. The role of stem cells in normal organs is known to preserve the capacities of an organ during a lifespan; however, its function in the TME is still uncertain. CSCs cooperate with several cells existing in the TME and based on the traded signals, these cells can be differentiated into any cell type (tumor heterogeneity) or stay in the inactive state for a long time, increasing the possibility of relapses [167].

Nano-system targeting strategies for CSCs are based on two methods, either targeting overexpressed receptors on CSCs or numerous signaling pathways participating in the growth and differentiation of CSCs. The cluster of differentiation-44 (CD44), which is a glycoprotein present on the surface of tumor cells, is overexpressed in CSCs and is mostly reported as a significant biomarker [168]. CD29, CD34, CD90, CD117, and Nestin are also some of the cellular markers overexpressed on cancer stem cells [169].

Ma et al. developed hyaluronic acid-linked nano-systems of mesoporous silica and reported a significantly more uptake in HeLa cell lines (with CD44 protein overexpression) in comparison to MCF-7 and L929 cell lines (low CD44-expression cell lines). The procedure was reported to be reliant on receptor-mediated endocytosis. Additionally, improved cytotoxicity of the drug camptothecin was observed due to encapsulation into these nanoparticles in comparison with the free drug [170].

Yi et al. produced glucose-modified gold NPs (Glu-AuNPs) via a two-step self-assembly. These developed Glu-AuNPs encapsulated siPLK1, an important gene involved in the cell cycle, to protect it from degradation. The nano-system reaches CSC targeting by recognizing and combining with its particular receptor glucose transporter 1 (GLUT1) overexpressed on the CSC surface. Due to the specific binding between the Glu ligands and GLUT1, the siPLK1-loaded Glu-Au NPs displayed increased cellular uptake as well as gene silencing efficiency and therefore enhanced anticancer activity both in the GLUT1-overexpressing MDA-MB-231 cell spheroids and MDA-MB-231 orthotropic tumor [171].

Kim et al. prepared aptamer-modified liposomes for delivery of the encapsulated doxorubicin to the cancer stem cells and breast cancer cells. Dually aptamer-modified nano-systems against CD44 on CSCs and MUC1 on breast cancer cells displayed higher cellular uptake and efficacy against metastasis of breast cancer stem cells in athymic nude mice [172].

Similarly, Ning et al. developed PEG-PCL-based NPs modified with an anti-CD133 antibody to deliver SN-38, a topoisomerase inhibitor. Enhanced targeting of CD133-positive (CD133þ) cells through receptor-mediated endocytosis was observed, and the same cytotoxic effect on CSCs as the siPLK1-loaded Glu-Au NPs was obtained [173].

Yu et al. reported increased cytotoxicity of doxorubicin (DOX) hydrochloride due to entrapment in hyaluronic acid-modified mesoporous silica nano-systems designed for targeted drug delivery to HCT-116 (CD44-overexpressed human colon cancer cells) [174].

### 4.3. Nanoparticles Targeting Cancer-Associated Fibroblasts

Fibroblasts are the regulators of the structure of normal tissues by ECM production and promoting tissue repair. Fibroblasts stay activated in cancerous tissue or “unhealed wounds” [175]. Cancer-associated fibroblasts (CAF) are a major part of the present cells of the tumor stroma, and they have a significant role in tumor growth due to controlling the tumor microenvironment. CAFs are responsible for the production of the major components of ECM consisting of types I, III, and V collagen and fibronectin, which limit the diffusion, and, therefore, the efficacy of cancer therapeutic agents inside the ECM [176,177,178]. Fibroblasts can be identified by two subtypes: the first group provides a supporting matrix for usual epithelial cells, and the second group, recognized as myofibroblasts, exists at the site of inflammation with a large quantity. The second subtype usually does not exist in normal and healthy tissues and is mostly present in the tumor environment [179]. Researchers have observed that drugs affecting these CAFs, along with cancer cells, have more efficacy in cancer treatment. These fibroblasts can provide an appropriate target for new chemical drugs. Vimentin, α smooth muscle actin (α-SMA), fibroblast activation protein (FAP), and ED-FN (ED splice variant of fibronectin) are some of the overexpressed biological markers on inactivated CAFs [180]. In the past years, many overexpressed markers on fibroblasts in the TME have been evaluated by using targeting ligands and have been found to have a therapeutic effect when used with additional chemotherapeutic agents [181].

Chen et al. [182] developed a nano-system modified with peptide FH, which is the specific ligand of Tenascin C, a protein that is mainly expressed by CAFs. These nanoliposomes were loaded with Navitoclax, which could stimulate apoptosis in CAFs. These targeted nanoparticles were shown to be effective for the eradication of CAFs and inhibition of tumor growth both in vitro and in vivo.

The M6P/hasnsulin-like growth factor II receptor (M6P/IGFIIR) is a transmembrane glycoprotein containing a huge extracellular domain. Diverse IGFIIR ligands, with and without mannose-6-phosphate (M6P), were studied, including granzyme B, renin, latent TGFβ1, thyroglobulin, proliferin, and retinoic acid or urokinase-type plasminogen activator receptor. The IGFIIR is upregulated in CAFs through hepatic carcinogenesis [183,184].

Beljaars et al. applied the M6P/IGFIIR for CAF-specific targeting by means of human serum albumin (HASA) as a carrier for the covalently linked M6P ligand. This system showed fast accumulation in the liver tissue and an improved cellular uptake in CAFs of fibrotic livers (approximately 50% of the intravenously injected dose). The system with the highest ratio of M6P to HSA (28:1) demonstrated the highest uptake in CAFs, proving that the amount of HSA-bound M6P is a significant factor for effective cell-specific targeting. In follow-up studies, M6P-HSA was conjugated into several drugs with anti-proliferative or antifibrotic effects, such as doxorubicin, mycophenolic acid, and 15-deoxy-∆12,14-prostaglandin J2 (15dPGJ2) [185].

In another study, Ji et al. prepared a peptide-assembled nanoparticle system consisting of CAF-targeted antibodies and doxorubicin (DOX). By means of CAF targeting and improved cellular uptake, the nano-system demonstrated high efficacy in the xenograft prostate tumor [186].

FAP-a is a protease on the surface of fibroblasts present in the TME. Based on the characteristics of this enzyme, Ji et al. designed a cleavable amphiphilic peptide (CAP) that is specifically cleaved by FAP-a. This CAP has the capability of being self-assembled and producing drug-loaded nanoparticles, which are easily broken by coming into contact with FAP-a and liberating the drug at the tumor site [187].

Anisamide is a ligand for sigma receptors, which are significantly overexpressed on fibroblasts. Miao et al. developed anisamide-modified nanoparticles loaded with the drug cisplatin and reported improved antitumor efficacy in the bladder cancer model, possibly because of affecting tumor cells along with CAFs [188]. The nanoparticle systems discussed in this review, their classification, specific characteristics, and further information are provided in Table 1.

### 4.4. Nanoparticles Targeting Endothelial Cells

It has been proven for several decades that vascular endothelial cells participate in sustaining suitable blood flow, nutrients, and several other present elements, even though they are a bit variable in different tissues. Physiological conditions of the TME also change roles, leading to malfunctions of the cells, which can help tumor growth. A defective endothelial monolayer of the present blood vessels of a tumor leads to a leaky vascular structure with an unusual blood current. Targeting endothelial cells is, therefore, of great importance for solving the problem of anticancer drug resistance. As mentioned before, angiogenesis is defined as the development of new blood vessels from already existing ones and is described as one of the cancer hallmarks [6]. Tumor progress relies on angiogenesis as it is an essential process for providing nutrients and oxygen, elimination of wastes, and metastatic capacity of cancer cells [189].

Vascular endothelial growth factor (VEGF) is a well-recognized angiogenesis stimulator overexpressing in the process of tumor development and metastasis [190].

Vascular endothelial growth factor receptor 2 (VEGFR-2) and neuropilin-1 (NRP-1) are two significant antiangiogenic targets. They are greatly expressed in vascular endothelial cells and some cancer cells. As a result, targeting VEGFR-2 and NRP-1 could be a possible antiangiogenic and anticancer strategy. A7R is a peptide capable of targeting VEGFR-2 and NRP-1 and degrading the link between vascular endothelial growth factor 165 (VEGF165) and VEGFR-2 or NRP-1 [191].

**Table 1:** Summary of targeted nanoparticles for cancer therapy discussed in this review.

Ref.	In Vivo and In Vitro Studies	Specific Characteristics and Results	Target	Nanoparticle System
Kim et al. [51]	Human ovarian A2780 carcinoma cells	DOX (weak base positively charged) released in inferior pH values quicker than physiologic pH due to the change of electrostatic and hydrophobic forces in the polymeric complex	Acidic pH of TME	Polymeric micelles (PMAA attached to PEO) loaded with DOX
Ding et al. [55]	Human A431 squamous carcinoma tumor-bearing nude mice	The acid-responsive hydrazine bonds of the polymer of nanoparticle made it a promising system for drug delivery in the TME	Acidic pH of TME	Multiblock polyurethane nanoparticle loaded with paclitaxel
Zhang et al. [57]	HeLa and 3T3 cell lines HeLa cells subcutaneously injected into nude mice	After 36 h of incubation, the DOX release from PLNPs-PAMAM-AS1411/DOX at pH 5.0 was around 60%, compared with a 10% release at physiological conditions.	Acidic pH of TME	PLNPs-PAMAM modified with AS1411 aptamer and loaded with DOX
Dominski et al. [58]	Human colon adenocarcinoma cell line HCT-116, human cell line MCF-7, normal human dermal fibroblasts-neonatal (NHDF-Neo)	The drug was released much faster at a lower pH in comparison with normal pH conditions by in vitro studies	Acidic pH of TME	Nano micelles consisting of biodegradable triblock copolymer poly(ethylene glycol)-b-polycarbonate-b-oligo([R]-3-hydroxybutyrate) loaded with doxorubicin and 8-hydroxyquinoline glucose- and galactose conjugate
Huang et al. [59]	BEL-7402 cells	The nanoparticles demonstrated efficiency for the delivery of intact DNA for in vivo gene transfection. The nanoparticles were internalized into intra-tumoral cells due to the upregulation of CPP, suggesting these nanoparticles as an effective gene delivery system	Acidic pH of TME	PEG-DGL nanoparticles modified with activatable cell-penetrating peptide (designated as dtACPP) sensitive to lower pH and MMP2 present in the TME
Li et al. [60]	A549 tumor cells and tumor-bearing mice	The developed nanoparticles with a size around 113nm demonstrated significant MRI and photothermal properties and were capable of drug release with the assistance of exogenous NIR.	Acidic pH of TME	Mesoporous silica nano-system covered with polydopamine-Gd3+ (PDA–Gd) adjusted by poly (2-Ethyl-2-Oxazoline) (PEOz) and loaded with DOX
Son et al. [61]	SW620 and DU145 cells, SW620 cells injected into 6-week-old mice	The polymeric micelles formed by a series of mPEG-bPCHGE polymers showed higher stability, encapsulation efficiency, and manageable release kinetics.	Acidic pH of TME	Nano micelles of mPEG-b-PCHGE containing an acetal group as a pH-responsive acetal cleavable linkage loaded with paclitaxel and Nile red dye
Dominski et al. [63]	Normal human dermal fibroblasts-neonatal (NHDF-Neo), colon carcinoma (HCT-116), and breast cancer (MCF-7)	The developed micelles with a size of about 55 nm were stable in physiological pH but degraded in acidic pH and demonstrated pH-dependent drug release behavior in vitro	Acidic pH of TME	Polymeric micelles of a synthesized diblock copolymer poly(ethylene glycol)-hydrazone linkage-poly[R,S]-3-hydroxybutyrate loaded with hydroxyquinoline glucose, galactose conjugates, and DOX
Wang et al. [64]	MCF-7, BxPC-3, and NIH/3T3 cells Female Balb/c-nude mice	In normal conditions of pH 7.4, the ligand was hidden in the PEG layer, while in the pH of the TME (6.5), the ligand was exposed and targeted liposomes. These liposomes showed the highest cytotoxicity and cellular uptake in vitro, tumor site accumulation, and best antitumor effect in vivo in comparison with non-sensitive liposomes	Acidic pH of TME	Tyrosine-modified poly-ethylene glycol monostearate liposome system encapsulating irinotecan
Liu et al. [65]	293 cells (a human renal epithelial cell line), HKC cells (a human renal tubular epithelial cell line), HeLa cells (a human epithelioid cervix carcinoma cell line), C6 cells (rat glioma cells), and Pc-12 cells (pheochromocytoma cells of the rat adrenal medulla) Glioma-bearing male CD-1 experimental mice	The developed nanoparticles simultaneously demonstrated efficiency in three types of therapy: chemodynamic treatment (CDT), chemotherapy, and photothermal therapy. This platform can be used as a multimodal synergistic cancer theranostic system	Acidic pH of TME	Fe–gallic acid (Fe–GA) nanospheres in combination with bovine serum albumin and encapsulating DOX
Du et al. [59]	MDA-MB-435s cells	This pH-responsive charge conversional nano-system promoted cellular uptake of Dox	Acidic pH of TME	PAMA–DMMA nanogels encapsulating Doxorubicin
Huo et al. [78]	Human epithelial HUVEC cell line cancerous HeLa cell line Breast and pancreatic tumor-bearing mice	The nanoparticles were degraded in the TME by MMP and enhanced the effect of radiation therapy	Hypoxia of TME	Tungsten oxide NPs (WO NPs)
				as sensitizers for radiotherapy modified by CCL-28 chemokine ligand and a matrix metalloproteinase cleavable peptide
Thambi et al. [79]	SCC7 cell line Nude mice bearing SCC7 tumor	A hydrophobically modified 2-nitroimidazole derivative was conjugated to the backbone of the nanoparticle responsible for the sustained release in normoxic conditions and burst release in hypoxia	Hypoxia of TME	Hypoxia-reactive carboxymethyl dextran nanoparticles containing doxurubicin
Son et al. [80]	SCC7 cells SCC7-bearing tumor athymic nude mice	The release degree of DOX increased by breakage of the azo bond in hypoxia conditions	Hypoxia of TME	Polymeric nanoparticles with carboxymethyl dextran and black hole quencher 3 encapsulating doxorubicin
Thambi et al. [81]	SCC7 cells	The hypoxia-sensitive polymeric micelles could preferentially release DOX under hypoxia conditions, proven by fluorescent imaging	Hypoxia of TME	Polymeric micelles with amphiphilic nature encapsulating DOX
Liu et al. [82]	K562 cell line (Leukemia cells) K562 tumor-bearing nude mice model	It was found that this nano-system can increase the sensitivity of the cells to the chemotherapeutic agent (danorubicin) and increase the intracellular density of DNR	Hypoxia of TME	PLGA-based nanoparticles modified with transferrin and loaded with danorubicin (DNR)
Zhu et al. [83]	EA.hy926 human umbilical vein cells HepG2 human hepatocellular carcinoma cells U87MG human glioma cells SGC-7901 gastric cancer cells MCF-7 human breast adenocarcinoma cells Nude mice bearing tumors	siHIF-1α cargo of the system was efficiently released in the hypoxic conditions of the TME. This system is also pH-responsive due to having hydrazone bonds. The intracellular delivery of siHIF-1α for gene silencing effects was enhanced significantly by this system	Hypoxia of TME	Hybrid quantum dots with a modified shell 2-deoxyglucose (DG)-polyethylene glycol (PEG) linked with the complex of lipoic acid, lysine, and 9-poly-d-arginine (LA-Lys-9R) by means of a hydrazone bond and a core of CdTe quantum dots
Abbasi et al. [84]	EMT6 breast tumor cell MDA-MB-231 cells BALB/c and SCID mice	Both systems enhanced the effect of radiotherapy when administered before radiation and also modulated the hypoxia of tumors significantly. Median host survival enhanced 3–5 fold	Hypoxia of TME	hybrid manganese dioxide (MnO_2_) nanoparticles (MDNP) consisting of hydrophilic terpolymer-protein or hydrophobic polymer-lipid for reoxygenating the TME by means of endogenous H_2_O_2_
Gao et al. [87]	Mice bearing 4T1 murine breast tumors	After IV injection, this nano-system oxygenates the whole TME and enhances the effect of radiotherapy	Hypoxia of TME	RBC-coated PLGA nanoparticles encapsulating PFC
Song et al. [89]	Murine breast cancer 4T1 cells Tumor-bearing Balb/c mice	Because of the high oxygen solubility of PFC, this nanoparticle can enhance the effect of DNA damage to cancer cells induced by X-ray	Hypoxia of TME	PEG nanoparticles containing PFC and decorated with TaO_x_ (an Xray absorber)
Song et al. [90]	4T1 murine breast cancer cells Tumor-bearing Balb/c mice	Due to the strong NIR absorbance of Bi_2_Se_3_. It can produce a strong photothermal effect as well as a radio-sensitizing effect. PFC is also responsible for releasing oxygen in the TME.	Hypoxia of TME	Bi_2_Se_3_ nanoparticles functionalized with PEG, encapsulating PFC and oxygen
Yin et al. [102]	MG63-osteosarcoma cells MG63 cell-bearing nude mice	The liposomes demonstrated high drug loading and stability under physiological conditions and degraded in the presence of reducing agents DTT and GSH. The disulfide bond containing liposomes showed high cellular uptake and internalization	Reductive environment of TME	Chotooligosaccharides (COS) Modified liposomes via a disulfide linker to cholesterol loaded with doxurubicin
Yin et al. [103]	MG63 osteosarcoma cells MG63 tumor-bearing nude mice	The liposomes with a size of around 110 nm demonstrated high cellular uptake in estrogen receptor-expressing osteosarcoma cells (MG63) and a rapid release of Dox due to the redox sensitivity	Reductive environment of TME	Estrogen-functionalized liposomes grafted with gluthathione-responsive chotooligosaccharides loaded with doxurubicin
Kumar et al. [104]	MCF-7, BT 474, and L929 cell line. Ehrlich’s ascites tumor cell line (EAT) (murine breast carcinoma) injected in Swiss albino mice	The nanoparticles demonstrated ~72% drug release at pH 5.5 in comparison with ~18% drug release at pH 7.4, and 91% tumor regression in Ehrlich ascites tumor (EAT) in comparison with free doxorubicin-treated mice	Reductive environment of TME	Folic acid and trastuzumab modified random multiblock copolymeric nanoparticles
Conte et al. [105]	A549 cells and spheroids	The nanoparticles were able to penetrate mucus and demonstrated high internalization ability in 2D and 3D models	Reductive environment of TME	PLGA-PEG nanoparticles containing disulfide bonds loaded with docetaxel
Wu et al. [106]	HeLa cells, human umbilical vein endothelial cells (HUVECs) Tumor-bearing female nude mice	MHPCNs−SS−PGA−FA nanoparticles demonstrated high drug loading capacity and efficient biodistribution in tumor sites as investigated with an MRI. This platform displayed synergistic photothermal/chemotherapy effects with decreased side effects	Reductive environment of TME	magnetic hollow and porous carbon nanoparticles (MHPCNs) covalently conjugated with cystamine dihydrochloride and capped with poly(γglutamic acid) (PGA) and Folic acid encapsulating DOX
Deng et al. [109]	HT-29 colorectal carcinoma cell line LNCaP metastatic prostate cancer cell line	The nanohydrogels demonstrated high cellular uptake and cytotoxicity due to the redox-responsive degradation and release of the oncolytic virus	Reductive environment of TME	Thiolated hyaluronic acid hydrogels encapsulating oncolytic viruses
Deng et al. [110]	RAW264.7 macrophage cell line	The nano-system demonstrated high internalization into macrophages, redox responsiveness, and high encapsulation efficiency for diverse proteins	Reductive environment of TME	Nanocapsules consisting of a triblock copolymer in the shell and thiolated hyaluronic acid in the core
Elgogari et al. [118]	P8, A6L, A32, P198, E3, P215, P10, JD13D patient-derived PDAC cell lines, and patient-derived pancreatic tumors	BPTES is a glutaminase inhibitor, and the nanoparticle system demonstrated a significant effect on pancreatic cancer models in combination with metformin therapy	Metabolic changes in TME	PLGA-PEG nanoparticles encapsulating BPTES
Gandham et al. [123]	human ovarian adenocarcinoma (SKOV-3) cell line	The surface-modified nanoparticles demonstrated increased permeability and cytotoxicity in the 3D multicellular model	Metabolic changes in TME	Liposomal nanoparticles encapsulating 3-BPA and modified with GE-11
Zhang et al. [124]	mouse pancreatic cancer cell line Pan-02 C57BL/6 mice	The liposomes demonstrated high efficiency in delivering 3-BPA to tumor cells overexpressing MCT1 and	Metabolic changes in TME	Liposomal nanoparticles functionalized with a pentapeptide encapsulating 3-BPA
Murty et al. [132]	human alveolar epithelial adenocarcinoma cells (A549) 6-week-old female nu/nu nude mice	The collagenase-modified nanoparticles demonstrated 35% higher accumulation within the tumor area	ECM network of TME	Gold nanoparticles labeled with collagenase
Villegas et al. [133]	human osteosarcoma cells (HOS) 3D collagen matrices housing HOS	The nano system showed a high penetration rate into the tumoral tissue model and a homogenous distribution and pH-responsive release due to the properties of the applied polymer	ECM network of TME	Polymeric nanocapsules encapsulating collagenase
Zinger et al. [134]	LSLKrasG12D/+;LSL-Trp53R172H/+ of pancreatic carcinomas Tumor-bearing C57BL/6 mice	the collagen component of the pancreatic tumor stroma was digested by collagenase encapsulated in nanoparticles. These nanoparticles, along with the administration of paclitaxel micelles, decreased the size of tumors by up to 87%	ECM network of TME	Nanoliposomes containing collagenase
Liu et al. [135]	4T1 tumor-bearing nude mice	Collagenase encapsulated nanoparticles contain acid-sensitive benzoic-imine organic linker that cleaves in hypoxic the TME and enhances the effect of chlorin e6 (Ce6)-loaded liposomes, which are applied for photodynamic therapy	ECM network of TME	Collagenase encapsulated Mn^2+^ based nanoparticles modified by PEG
Loskutov et al. [138]	Human astrocytes isolated from the human cortex Tumor-bearing immunodeficient male mice	LPA signaling was limited significantly by means of this nano-system, and tumor progression was inhibited.	ECM network of TME	PLGA-PEG nano-system encapsulating small molecule Ki16425 (an LPA signaling inhibitor)
Sun et al. [141]	HUVEC and A549 lung cancer cells J774A.1 macrophage cells Lung tumor-bearing BALB/c mice	It was observed that the encapsulated paclitaxel was efficiently released in the high concentration of MMP in the tumor microenvironment and this nano system was efficient in treating lung cancer	ECM network of TME	Methoxy-poly(ethylene glycol)-poly(lactic acid) (MPEG-PLA) nanoparticles modified with a multitargeting peptide-LinTT1(MMP sensitive) and a cellpenetrating peptide-TAT encapsulating paclitaxel
Anajafi et al. [142]	BxPC-3 and AsPC-1 cells (human pancreatic adenocarcinoma, ATTC) and the 3D cultures	The polymerosomes taking advantage of the redox sensitivity and active targeting by means of MMP-7, demonstrated high penetration and shrinkage of the spheroids up to 49% in comparison to the normal cells	ECM network of TME	Polymeric vesicles surface modified with matrix metalloproteinase-7 (MMP-7) encapsulating curcumin and doxorubicin
Chen et al. [147]	4T1 murine breast cancer and CT26 colorectal cancer cell Tumor-bearing BALB/c mice	Great anti-tumor efficacy was absorbed by applying these nanoparticles, followed by anti-CTLA4 therapy. This nanoparticle combined therapy encapsulating both NIR heaters and immune-adjuvant TLR agonists can stimulate vaccine-like immune responses	Tumor-associated immune cells	PLGA nanoparticles encapsulating Indocyanine green (photothermal agent) and imiquimod (R837), a Toll-like-receptor-7 agonist
Conde et al. [158]	A549-luciferase-C8 human lung adenocarcinoma cells lung cancer orthotopic murine model (BALB/c nude)	The nanoparticles demonstrated targeted delivery to murine lung TAMs and delivery of siRNA to those cells and to lung cancer cells. Due to the hybrid approach (silencing the VEGF gene), this system showed significant efficacy in inhibiting tumor progression.	Tumor-associated immune cells	RNA interference (RNAi)-peptide gold nanoparticles surface functionalized with M2 peptide and thiol-siRNA-Alexa Flour 488
Schmid et al. [159]	Murine T cells B16 melanoma cells inoculated in six-to-ten-week-old C57BL/6 mice	The nanoparticles were capable of targeting T cells and delivery of the payload resulting in tumor growth delay and enhanced survival of tumor-bearing mice	Tumor-associated immune cells	PLGA-based nanoparticles surface modified with PD-1 antibodies encapsulating aTLR7/8 agonist or inhibitors of TGFBR1
Yang et al. [160]	Primary CD8^+^ T cells Female C57Bl/6 mice 6–8 weeks	Targeted nanoparticles displayed a 40-fold increased uptake in CD8+ T cells in comparison with the non-targeted nanoparticles, and they showed high efficiency in a cancer vaccine model	Tumor-associated immune cells	Antibody-modified amphiphilic organic ligand-protected gold nanoparticle containing a small molecule TGF-β inhibitor
Sharma et al. [162]	RAW264.7 cells CCL-110 human fibroblast cell line 4T1 tumor-bearing female Balb/c mice	The nanoparticles demonstrated high encapsulation, targeting, and an eight-fold increase in cell death in comparison with free drug and blank nanoparticles	Tumor-associated immune cells	PLGA NPs functionalized with the LyP-1 peptide encapsulating clodronate
Zhu et al. [163]	murine J774A.1 macrophage cells	These nanoparticles can efficiently target TAMs in the tumor microenvironment. PEG shedding of the nano-system is also responsible for delivery in an acidic condition of the TME	Tumor-associated immune cells	PLGA-PEG nanoparticles modified with mannose
	B16-F10 mouse melanoma tumors in C57BL/6 mice			
He et al. [164]	J774A.1 cells MCF-7 cells	By dual targeting with MCMC (for mannose ligands) and HA, this nano-system upregulates proinflammatory cytokines and shifts macrophages in M1 polarity	Tumor-associated immune cells	Mannosylated carboxymethyl chitosan (MCMC)/hyaluronan (HA) nanoparticles for delivery of CpG oligodeoxynucleotides (ODN)
Qiu et al. [165]	RAW264.7 macrophage S180 murine sarcoma cell line Kunming male mice	IBR is a Bruton’s tyrosine kinase (BTK) inhibitor, and BTK is overexpressed on TAMs. The nano-system delivered IBR to the tumor microenvironment efficiently and significantly reduced tumor growth.	Tumor-associated immune cells	Amphiphilic egg phosphatidylglycerol (EPG) nanoc-omplex modified with sialic acid (SA)–stearic acid conjugate encapsulating Ibrutinib (IBR)
Ordikhani et al. [166]	B16-F10 murine melanoma model Female C57BL/6, PD-1^−/−^ LT-α^−/−^ and BALB/c mice (7–9 weeks old)	Administration of high dose of anti–PD-1 NPs in mice resulted in increased mortality in comparison with those treated with free anti-PD-1 antibody because of the overactivation of T cells. Further modification of the anti-PD-1 NPs dosage resulted in less toxicity and higher antitumor effect of nanoparticles	Tumor-associated immune cells	Anti–PD-1 antibody encapsulated in PLGA nanoparticles
Ma et al. [170]	HeLa, MCF-7 and L929 cell lines	This nano-system demonstrated significant efficacy against CD44 overexpressing cancer cells	Cancer stem cells	Mesoporous silica linked with hyaluronic acid encapsulating camptothecin
Yi et al. [171]	Human breast cancer MDA-MB-231 cells Tumor-bearing NOD/SCID mice or BALB/c nude mice (female, eight weeks old)	The nanoparticles displayed high internalization into glucose transporter 1- overexpressing breast CSCs. These nano-systems stimulated gene silencing in a CSC-rich in vivo model	Cancer stem cells	Glucose-linked unimer polyion complex-assembled gold nanoparticles for targeted siRNA delivery
Kim et al. [172]	The human breast cancer cell line MCF-7 (MUC1/CD44 positive) human hepatocellular carcinoma cell line HepG2 (MUC1/CD44 negative) and their 3D cultures	Dual-aptamosomes had more cytotoxic effects on both CSCs and cancer cells in comparison to non-targeted liposomes and had shown an inhibitory effect against metastasis of breast CSCs and cancer cells in nude mice.	Cancer stem cells	Anti-MUC1/CD44 Dual-Aptamer-Conjugated Liposomes containing doxorubicin
Ning et al. [173]	Human colorectal cancer cell lines HT-29, SW620 and HCT116 cells HCT116-bearing female nude mice at the age of 4 weeks	The nanoparticles were efficiently targeted and internalized to CD133 overexpressing HCT116 cells and demonstrated high cytotoxicity. Immunohistochemistry results showed a reduction in CD133 expression in these cells after treatment with these nanoparticles.	Cancer stem cells	PEG−PCL-based nanoparticles modified with Anti-CD133 antibody encapsulating a topoisomerase inhibitor (SN-38)
Yu et al. [174]	HCT-116	This nano-system significantly increased cellular uptake via HA receptor-mediated endocytosis in CD44 positive cell line (HCT-116 cells)	Cancer stem cells	Hyaluronic acid (HA) modified mesoporous silica nanoparticles encapsulating doxurubicin
Chen et al. [182]	LX-2, Hep G2 cells Tumor-bearing BALB/c nude mice	The antitumor efficacy of the nanoparticles was significantly higher than the free navitoclax and unmodified nanoliposomes	CAF	Navitoclax-loaded nanoliposomes modified with peptide FH (ligand of tenascin C, mainly expressed by CAFs)
Ji et al. [186]	CAFs, PC-3 (a prostate cancer cell line), and human umbilical endothelial cells (HUVECs) CAFs and PC-3 cells bearing nude mice	The mAb-modified PNPs demonstrated higher cellular uptake by CAFs, higher tumor penetration and less side effects of the encapsulated drug compared to non-modified PNPs	CAF	Polymeric nanoparticles (PNP), with a hydrophobic cholesterol core and a hydrophilic cationic R9 peptide shell modified with fibroblast activation protein-α (FAP-α) targeting antibody encapsulating doxurubicin
Ji et al. [187]	PC-3 (a prostate cancer cell line) Human umbilical endothelial cells (HUVECs), CAFs MCF-7 breast tumor Mia-paca-2 pancreatic tumor	The CAP-NPs showed promising antitumor efficacy for solid tumor models (breast and pancreatic tumors).	CAF	A nanoparticle consisting of cleavable amphiphilic peptide (CAP) responsive to fibroblast activation protein-a (FAP-a) expressed on CAFs encapsulating doxorubicin
Miao et al. [188]	Human bladder transitional cell line (UMUC3) Mouse embryonic fibroblast cell line (NIH 3T3) Bladder tumor Balb/C nude mice	It was observed that intravenous injection of these nanoparticles, along with cisplatin nanoparticles, inhibited tumor growth in the early and late stages of bladder cancer.	CAF	Liposome-protaminehyaluronic acid NP (LPH-NP) encapsulating siRNA against Wnt16 (siWnt) affecting cancer-associated fibroblasts
Du et al. [192]	Human hepatoma cells (HepG2) primary human umbilical vein endothelial cells (HUVECs) Tumor-bearing mice	The nanoparticles demonstrated normalizing the vascular structure and function of tumor blood cells. When loaded with Gem, the antitumor efficacy increased significantly.	Endothelial cells	Lipid derivative conjugates (LGCs) nanoparticles made of low molecular weight heparin (LMWH) and gemcitabine (Gem)
Cao et al. [193]	MDA-MB-231 and MCF-7 human breast cancer cell lines Tumor-bearing BALB/c nude mice	A7RC increased the targeting efficacy of nanoparticles significantly in high NRP-1 expressing cells of breast tumors, and nanoparticle accumulation and cellular uptake increased dramatically	Endothelial cells	Nanoliposomes modified with A7R-cysteine peptide (A7RC) encapsulating paclitaxel
Lu et al. [194]	Human brain microvascular endothelial cells (HBMECs) and C6 glioma cell line Wistar rats and New Zealand white rabbits.	The developed nanoparticles displayed high release (79.5%) in the reduced pH of the in vitro tests (pH = 5.5). Modification of the nanoparticles with RGD enhanced the cytotoxicity effect in in vitro BBB model due to increased uptake by C6 cells.	Endothelial cells	RGDyC/PEG co-modified PAMAM nanoparticles encapsulating arsenic trioxide
Murphy et al. [195]	Human umbilical vein endothelial cells (HUVECs) M21L-GFP mouse melanoma cells (integrin αvβ3 negative) iv injected mice	αvβ3 mediated drug delivery demonstrated a dramatic (15-fold) antimetastatic activity and a decrease in the side effects	Endothelial cells	RGD-modified polymeric nanoparticles encapsulating doxurubicin
Eloy et al. [196]	DU145 and PC3 cell lines	Modified nanoliposomes had more targeting efficacy on EGFR overexpressing cell line (DU145) in comparison with PC3	EGFR	Nanoliposomes modified with anti-EGFR antibody containing docetaxel
McDaid et al. [197]	HCT116, A549, HKH-2, HCC827, PANC-1 cell lines	Cetuximab acts as a targeting agent for EGFR and demonstrated significant effects in pancreatic tumors	EGFR	PLGA nanoparticles modified with Cetuximab (CTX) encapsulating camptothecin
Aggarwal et al. [198]	MIA PaCa-2 (human pancreatic carcinoma)	This nano-system is a promising platform for EGFR- positive cancers therapy	EGFR	PLGA-PEG nanoparticles modified with EGFR antibody and loaded with Gemcitabine

Du et al. [192] produced a tumor vessel normalization prompting nano-system modified with low molecular weight heparin (LMWH) and gemcitabine (Gem) loaded with paclitaxel. LMWH blocks the VEGF signaling pathway by inhibiting VEGF from binding to its ligand on endothelial cells. This nanomedicine degrades in an acidic TME and releases Gem and paclitaxel.

Cao et al. developed A7R-cysteine peptide (A7RC) surface-adjusted paclitaxel liposomes (A7RC-LIPs) for targeted drug delivery and limiting the tumor progress and angiogenesis at the same time. The results proved that A7RC peptides are capable of enhancing the vesicle uptake by MDA-MB-231 cells, resulting in improved cytotoxicity in vitro and greater vesicle accumulation in MDA-MB-231 xenografts in vivo [193].

Several other biological markers are also expressed in endothelial cells. For instance, av b3 is a subtype of integrin present in almost all types of tumors, as well as tumstatin and VEGF. Integrin is the class of glycoproteins that regulates cell-to-cell and cell-to-ECM interactions [199].

Arginine-glycine-aspartic acid (RGD) is the natural ligand of av b3 integrin and is commonly used by researchers for imaging, diagnostic, and treatment purposes. This ligand distinguishes integrin heterodimers between the αV unit (CD51) and the β3 unit (CD61), which is a receptor for vitronectin on the surface of TECs and is involved in angiogenesis.

Lu et al. produced a cRGD-modified PAMAM dendrimer encapsulating arsenic trioxide, which is a conventional Chinese medicine for treating cancer. This nano-system was capable of arresting the cell cycle in the G2/M phase and demonstrated restricted proliferation in glioma cells [194].

Murphy et al. observed a 15- fold rise in the antimetastatic capacity of the drug doxorubicin loaded in RGD-modified nanoparticles via targeting the av b3 integrin [195]. This ligand was evaluated with other metallic nano-systems for targeting along with producing hyperthermia or improving the radiosensitivity of tumor cells [200].

**Figure 5:**
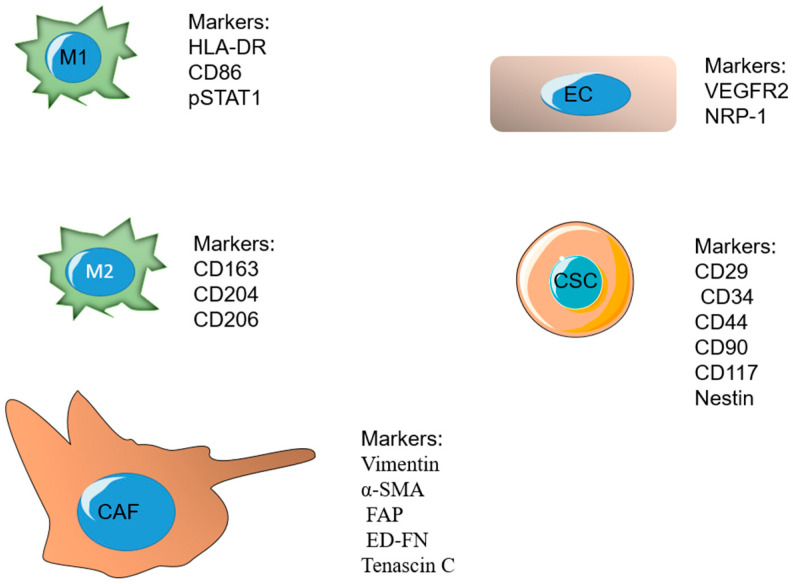
Some of the important present cells in the tumor microenvironment and their specific markers.

Figure 5 represents some of the present cells in the TME and their markers, which could be used for developing specific targeted nanoparticles.

### 4.5. Nanoparticles for Targeting Epidermal Growth Factor Receptor

The epidermal growth factor receptor (EGFR), also called HER-1 or ErbB1, along with the proteins HER-2/ErbB2, HER-3/ ErbB3, and HER-4/ErbB4, creates a group of tyrosine kinase type receptors present in cell cytoplasmic membrane [201].

By means of the ligand, the EGF receptor goes through configurational alterations that lead to its dimerization with the other proteins of the ErbB group or with a homologous receptor and later phosphorylation of tyrosine remains of the intracellular domain [202].

It has been reported the direct or indirect connection of EGFR with the pathogenesis of some of the tumors with the highest frequency and mortality rates worldwide, such as colorectal, lung, and breast cancer [203]. Therefore, several antibodies for EGFR have been developed and approved for cancer treatment, such as cetuximab (CTX), nimotuzumab, panitumumab, and necitumumab, and many nanoparticle formulations have been functionalized with these antibodies with the aim of better targeting.

Considering that many small molecules applied as cancer therapeutic agents are hydrophobic, which limits their delivery, EGFR-targeted nanoparticles can have a dual function for increasing the drug’s solubility and stimulating tumor-targeting properties [204].

Eloy et al. developed immune liposomes containing DTX and functionalized them with an anti-EGFR antibody. The cytotoxicity assessments were done on DU145 (with high expression of EGFR) and PC3 (with low EGFR expression) prostate cancer cell lines. It was revealed that DTX containing immunoliposomes functionalized with EGFR antibody had more efficiency against DU145 with higher expression of EGFR, emphasizing the role of EGFR as a target for cancer therapy [196].

McDaid et al. developed camptothecin-containing nanoparticles functionalized with cetuximab. The efficacy of these nanoparticles was studied in many EGFR-positive cell lines of lung, colon, and pancreatic cancers. It was observed that CTX-functionalized nanoparticles were more efficient against the cell lines with EGFR overexpression [197].

In another study, Aggarwal et al. formulated PLGA-PEG nanoparticles containing gemcitabine functionalized with anti-EGFR antibodies and studied the response of human pancreatic cells (MIA PaCa-2) [with high expression of EGFR] to these nanoparticles. The cell uptake studies by confocal microscopy and MTT assay revealed the specific targeting of these nano-systems to the mentioned cell line [198].

In addition to all these targets, there are several other biomarkers on the cells corresponding to one or more types of cancer, and many more are to be discovered by researchers. These markers may be upregulated on the cancer cells or other cells present in the TME and can be used for targeting the desired nanoparticles in the cancer environment.

## 5. Conclusions

Nanoparticles have provided a promising platform for cancer therapy in the past decades because of their specific characteristics and modifiable features. Nanoscale systems have many advantages over traditional drug delivery systems as they can be designed with different features, such as size, zeta potential, loaded cargo, and targeting ligands. The formulation of nanoparticles for cancer therapy can take advantage of many targeting mechanisms at cellular and molecular levels. The tumor microenvironment (TME) has many specific characteristics; it is hypoxic, with a significant amount of reducing agents, and has an acidic pH. The extracellular matrix of the TME is more dense and rigid, with different concentrations of molecules compared with normal ECM. Nanoparticles can be designed to release cargo in these conditions or be modified to prevent drug resistance for other therapies, such as radiotherapy and photodynamic therapy.

Nanoparticles can also be designed for the delivery of therapeutics to the cells present in tumors, such as tumor cells and various types of immune cells. They can be modified with the ligands of the cell targets or target the specific features of the present cells in the TME.

One of the most significant problems of cancer therapy is the limited information about the behavior of the immune system in the tumor development process. The immune system consists of complex mechanisms, and the effect of elaborating on each component of this system is not totally clear to scientists. The other reason relies on the heterogeneity of tumors, which makes the therapy path even more difficult. The immune systems of different individuals do not respond the same way to the same cancer therapies. Due to the complexity of the cancer progression process, it seems reasonable that nanoparticles employing multiple approaches for drug delivery would be more successful in cancer therapy. For instance, recently, many nanoparticles have been designed that can release their cargo in the specific conditions of the TME (hypoxia, redox, metabolic changes, etc.) and are modified on their surface by different ligands of the present cells in the TME, such as targeting antibodies, aptamers or targeting peptides. Employing multiple targeting approaches can lead to more effective nanoparticles with less chance of developing tumor resistance and therapeutic failure, leading to more clinically efficient cancer treatment.

## Data Availability

Not applicable.

## Abbreviations

ATX	autotaxin enzyme
α-SMA	α smooth muscle actin
BDO	1,4-butanediol
BHQ3	black hole quencher 3
BPTES	bis-2-(5-phenylacetamide-1,2,4-thiadiazol-2-yl) ethyl sulfide
CAF	cancer-associated fibroblasts
CAP	cleavable amphiphilic peptide
CART	chimeric antigen receptor T cell immunotherapy
CM-Dex	carboxymethyl dextran
CSC	cancer stem cells
CTLA-4	cytotoxic T-lymphocyte-associated antigen-4
CTX	cetuximab
DDS	drug delivery system
DG	deoxyglucose
DOX	doxorubicin
EC	endothelial cells
ECM	extracellular matrix
EPO	erythropoietin
EPR	enhanced permeability and retention
EMT	epithelial-mesenchymal transition
FAP	fibroblast activation protein
FGF	fibroblast growth factor
GLUT-1	glucose transporter-1
HR-NP	hypoxia responsive nano particles
HIF	hypoxia-inducible factor
LDI	L-lysine ethyl ester diisocyanate
LPA	Lysophosphatidic acid
LPC	lysophosphatidylcholine
MDSC	myeloid-derived suppressor cells
MMP2	matrix metalloproteinase-2
NK	natural killer
NP	nanoparticle
NIPAM	poly(N-isopropylacrylamide)
NIR	near-infrared
PAA	peroxy acetic acid
PBAA	poly(2-n-butylacrylic acid)
PbAE	poly beta-amino ester
PCL	polycaprolactone
PD-1	programmed death-1
PDAC	pancreatic ductal adenocarcinoma
PDL-1	programmed death-ligand-1
PEAA	polyethylacrylic acid
PEO	polyethylene oxide
PEG	polyethylen glycol
PFC	perfluorocarbon
PGA	poly(glycolic acid)
PMAA	poly methacrylic acid
PPAA	poly propylacrylic acid
PTT	photothermal therapy
PTX	paclitaxel
RB	retinoblastoma
QD	quantum dots
RBC	red blood cell
ROS	reactive oxygen species
RGD	Arginine-glycine-aspartic acid
SMA	smooth muscle actin
TAM	tumor-associated macrophages
Ta Ox	13tantalum oxid
TIL	tumor-infiltrating lymphocytes
TME	tumor microenvironment
TP53	tumor protein53
VEGF	vascular endothelial growth factor
VEGFR	vascular endothelial growth factor receptor
